# Design and Application of a Phenanthroline-Based Covalent Triazine Framework with Cu(I) for the Heterogeneous Synthesis of 2‑Aminobenzothiazoles

**DOI:** 10.1021/acsami.6c05338

**Published:** 2026-05-04

**Authors:** Jorge Vega, Maria Jose Capitan, Jesus Alvarez Alonso, Alberto Fraile, José Alemán

**Affiliations:** a Organic Chemistry Department, Science Faculty, 16722 Universidad Autónoma de Madrid, Madrid 28049, Spain; b Departamento de Física de la Materia Condensada, 16722 Universidad Autónoma de Madrid, Madrid 29049, Spain; c Física de sistemas crecidos con baja dimensionalidad, Universidad Autónoma de Madrid, Unidad Asociada al CSIC por el IEM, DP, Madrid 29049, Spain; d Instituto de Ciencia de Materiales "Nicolás Cabrera", 16722 Universidad Autónoma de Madrid, Madrid 28049, Spain; e Instituto de Física de la Materia Condensada IFIMAC, 16722 Universidad Autónoma de Madrid, Madrid 28049, Spain; f 16379 Instituto de Estructura de la Materia IEM-CSIC, Madrid 28006, Spain; g Institute for Advanced Research in Chemical Sciences (IAdChem), 16722 Universidad Autónoma de Madrid, Madrid 28049, Spain

**Keywords:** heterogeneous catalysis, covalent triazine framework (CTF), 1,10-phenanthroline, copper(I) catalyst, 2-aminobenzothiazole, C−S bond formation

## Abstract

Catalyst design is essential for advancing sustainable chemistry, particularly through heterogeneous systems that offer recyclability and environmental benefits. Herein, we report the synthesis and application of a novel covalent triazine framework (CTF) incorporating 1,10-phenanthroline units, which serve as coordination sites for Cu­(I) ions. The resulting heterogeneous catalyst, **Cu@Phen-CTF**, was prepared through postsynthetic metalation and fully characterized by Fourier-transform infrared, Raman, solid-state nuclear magnetic resonance, scanning electron microscopy/energy-dispersive X-ray, transmission electron microscopy, X-ray photoelectron spectroscopy, and Brunauer–Emmett–Teller surface area analysis, confirming successful Cu­(I) incorporation and structural integrity. This catalyst was applied to the tandem cyclization of 2-iodoanilines with isothiocyanates to access pharmacologically relevant 2-aminobenzothiazole derivatives. The **Cu@Phen-CTF** system showed excellent catalytic activity under mild conditions (50 °C, toluene, 72 h), affording high yields and a broad substrate scope. Furthermore, the material demonstrated good thermal stability, negligible leaching, and high recyclability, maintaining performance across multiple catalytic cycles.

## Graphical abstract

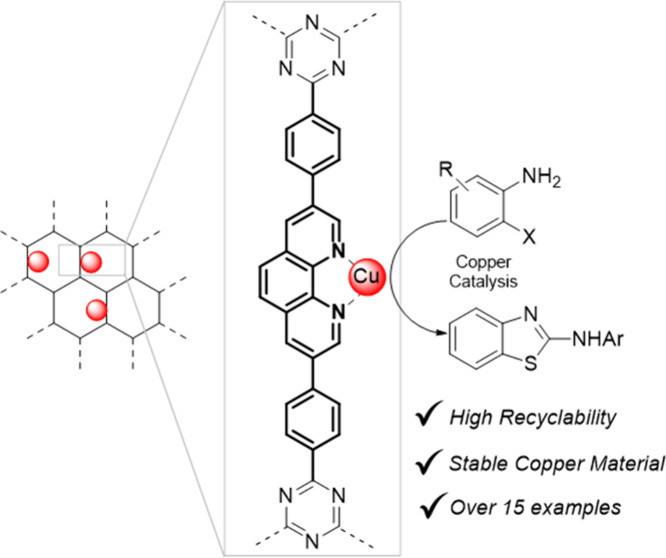

## 1 Introduction

Catalysts play a crucial role in advancing sustainable chemistry.^1^ As a result, significant efforts have been dedicated to their development, with a particular focus on heterogeneous catalysis due to its environmental benefits and recyclability.^2–4^ Its advantages stem from easy separation and recovery as well as suitability for continuous reactor operations. One strategy for designing heterogeneous catalysts involves immobilizing them within material frameworks such as carbon nanotubes,^5–8^ covalent organic frameworks,^9–11^ or metal–organic frameworks,^12^ among others.^13–15^ A wide range of supports are available for this purpose, but selecting the right support is essential. The choice not only ensures the sustainable benefits of heterogeneous catalysts but also introduces additional features that can enhance the catalytic performance or modify the selectivity of a given transformation.

Heterocyclic compounds and their derivatives have attracted increasing attention, owing to their significant relevance in medicinal chemistry. Within this class, 2-aminobenzothiazole-based frameworks stand out due to their structural versatility and straightforward synthetic accessibility, making them valuable platforms in both synthetic organic chemistry and biological applications, largely driven by their pronounced pharmacological properties.^16^ In this context, 2-aminobenzothiazoles (right, Figure 1a) are defined as bicyclic systems consisting of a benzene ring fused to a thiazole moiety, which have been widely recognized for their potential in pharmaceutical and agrochemical applications.^17–22^

**Figure 1:**
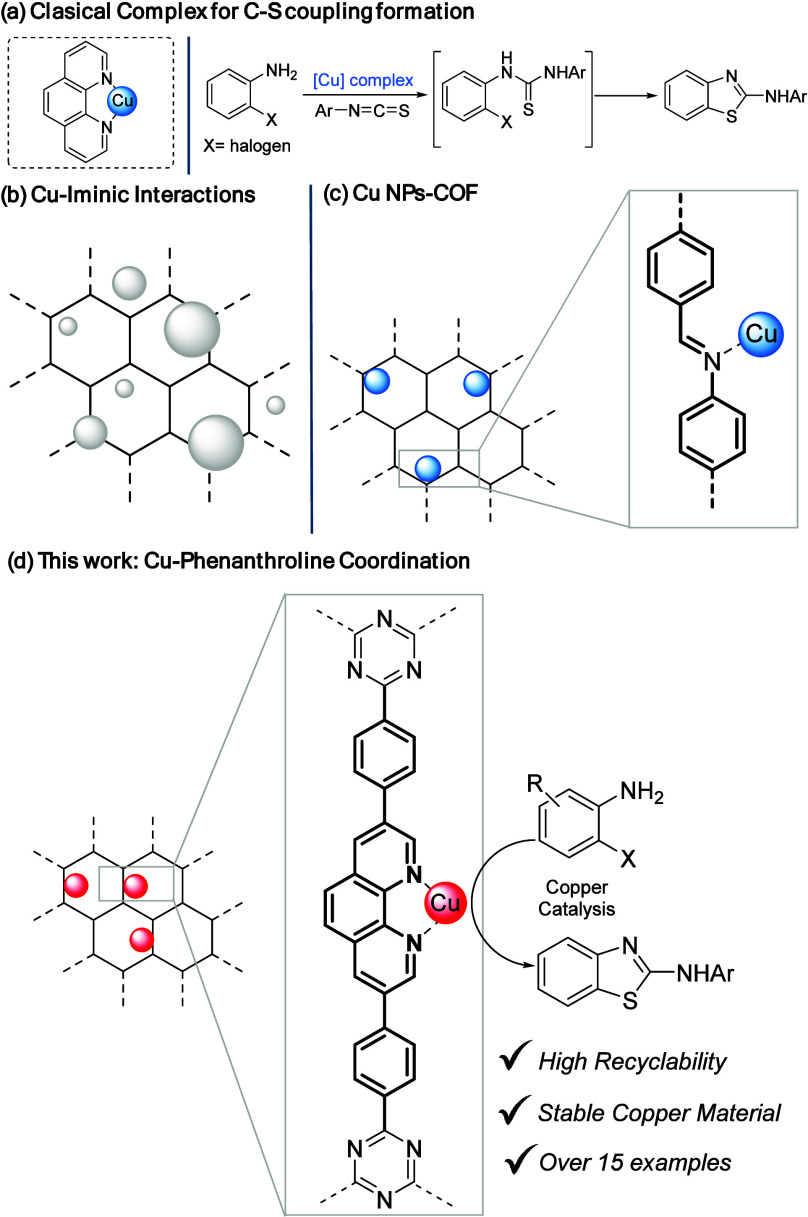
(a) Previous work in the homogeneous synthesis of bioactive 2-aminobenzothiazole derivatives. (b, c) Others use COP material with copper. (d) New design and application of Cu phenanthroline material.

One of the best methods to access these 2-aminobenzothiazol derivatives is the use of isothiocyanates as a precursor, having gained great attention due to their easy availability and high reactivity.^23,24^ In 2009, Wu et al. described the efficient and versatile homogeneous approach to 2-aminobenzothiazole scaffolds by a copper­(I)-catalyzed reaction of 2-iodobenzenamine with isothiocyanate under mild conditions and in the presence of 1,10-phenanthroline as the best ligand (right, Figure 1a).^23^ This straightforward approach led to a broad variety of 2-aminobenzothiazole derivatives with a great range of substituents both in the aromatic ring and at the amine group (aliphatic and aromatic residues are tolerated) in good to high yields. However, despite the good results obtained, the handicap of this homogeneous version approach is the impossibility of recovering the catalyst once the reaction is completed, which, from the point of view of sustainability, is a big problem. That is why the implementation of this process in its heterogeneous version is a very desirable issue, because it would allow the separation and recovery of the catalytic system from the reaction medium. In addition, the immobilization and isolation of catalytically active sites prevent the agglomeration, leakage, and deactivation of the metal catalyst during the reaction.^25^ In this regard, 1,10-phenanthroline (Phen) is a well-established bidentate chelating ligand for transition metal ions, which has historically played a central role in the advancement of coordination chemistry^26,27^ and continues to attract significant interest as a versatile precursor in organic, inorganic, and supramolecular chemistry (Figure 1a, left). Structurally, Phen is a rigid, planar, and hydrophobic heteroaromatic system with an electron-deficient character, featuring two inward-oriented nitrogen donor atoms positioned in close proximity, thereby preorganizing the ligand for strong and entropically favorable metal coordination. These structural characteristics govern its coordination behavior toward metal centers. Additionally, the σ-electron-deficient nature of this class of ligands renders them effective π-acceptors, enabling stabilization of metal ions in lower oxidation states. The combination of these structural and electronic features in Phen has led to extensive investigation of its metal complexes, particularly in relation to their catalytic,^28–30^ redox,^31^ photochemical, and photophysical behavior.^32–34^ Consequently, Phen-based coordination motifs have also been widely explored as key components in the development of efficient luminescent materials and even photoswitchable molecular systems.^35,36^

In this sense, covalent organic polymers (COP) have appeared as promising hosts to immobilize metals to be used as heterogeneous catalytic systems. COPs are porous, cross-linked materials with robust covalent bonds that confer high chemical and thermal stability, along with remarkable crystallinity and specific surface area.^37,38^ Moreover, these materials are characterized by notable attributes, including high thermal stability, low toxicity, reduced density, tunable porosity, and a high degree of functional diversity and adaptability. Their structural design enables the incorporation of specific functional groups, which has driven their development as catalysts in various applications or allows the introduction of metal cations into predefined active sites of the organic network.^25^ Specifically, incorporation of metal centers such as palladium,^39^ iridium,^40^ etc. in their structure allows their use in photocatalytic reactions such as the hydrogen generation via water splitting, organic oxidation, and pollutant degradation through photoinduced processes. Additionally, these metalated materials have gained increasing relevance in C–C and C–N coupling reactions, where the stability of the organic support allows catalyst recyclability and prevents metal leaching. Regarding cross-coupling reactions, such as Suzuki–Miyaura, Sonogashira, and Heck, COFs and CTFs functionalized with palladium and copper have exhibited catalytic activity comparable to homogeneous systems, with the added advantage of easy recovery.^25^ Similarly, in Buchwald–Hartwig amination, nickel- and palladium-based COFs have demonstrated high efficiency, offering a sustainable and economically viable alternative to traditional catalysts.^41^ Although in these cases, different materials have been widely used for the formation of C–C, C–O, and C–N bonds,^25,41^ the formation of C–S bonds using COPs with copper has been scarcely explored. Therefore, the synthesis of a material with an appropriate ligand with copper, in this case, phenanthroline, would be desirable in order to carry out the formation of a C–S bond and apply it to the synthesis of bioactive compounds of interest such as 2-aminobenzothiazole scaffolds.

The superficial functionalization of COFs and related porous materials with highly active Cu(0) nanoparticles (Figure 1b)^42–45^ and Cu­(II) salts (Figure 1c)^46–49^ has been extensively studied. However, achieving the coordination of single-atom Cu­(I) centers using COFs remains a more challenging objective, especially considering the low stability of Cu­(I) and its easy oxidation to Cu­(II), so there are few examples.^50–52^

Therefore, incorporating it into a material that protects it from oxidation would be advisible. Thus, copper-incorporated COFs are typically reported with imine linkages, where copper coordinates with the imine bonds (Figure 1c).^53–57^ However, it is well-known that the robustness of the imine-Cu bond is not very strong, making leaching relatively easy and hindering reuse and recycling of the material. In this work, we have designed a new triazine-based material incorporating a phenanthroline unit, to which we have added a Cu­(I) catalyst (Figure 1d). Additionally, we conducted a study on the structural scope of the reaction and its recyclability.

## 2 Experimental Section

### 2.1 Synthesis of **Cu@Phen-CTF**

#### 2.1.1 Synthesis of 4,4′-(1,10-Phenanthroline-3,8-diyl)­dibenzonitrile (**1**)^58^

3,8-Dibromo-1,10-phenanthroline (400 mg, 1.18 mmol), cyanophenylboronic acid (520 mg, 3.54 mmol), potassium carbonate (980 mg, 7.10 mmol), and Pd­(PPh_3_)_4_ (272 mg, 0.24 mmol) were placed in a Schlenk flask fitted with a magnetic stirrer. The flask underwent three vacuum-argon cycles. Subsequently, 10 mL of 1,4-dioxane and 2 mL of predegassed distilled water were added. The resulting mixture was heated at 100 °C for 72 h. Following this, the solvent was removed via evaporation under reduced pressure. The remaining solid was ground with distilled water, filtered, and then dissolved in 250 mL of hot CHCl_3_. The solution was rapidly filtered through a plug of Celite, washed with CHCl_3_, and concentrated by evaporation under vacuum. Finally, it was rinsed with cold acetone and dried under vacuum, yielding 70% product **1**. The reported nuclear magnetic resonance (NMR) data matches with what is described in the literature.^58^

#### 2.1.2 Synthesis of **CTF-Phen** ^58^

In a 25 mL sealed tube fitted with a Teflon screw cap, monomer **1** (200 mg, 0.520 mmol) was dispersed in 5 mL of dichloromethane under an ultrasound bath for 10 min. Then, the solution was cooled in an ice bath and 2 mL of trifluoromethanesulfonic acid was added dropwise. The flask was sealed, and the mixture was heated to 100 °C for 24 h. After cooling down to room temperature, 20 mL of a mixture of EtOH–water (1:1) was added dropwise with gentle stirring. The yellow solid was isolated by centrifugation and repeatedly washed with EtOH–water (1:1) until neutral pH was reached. Then, the material was finally washed with acetone and diethyl ether and dried under vacuum for 24 h.

#### 2.1.3 Synthesis of **Cu@Phen-CTF**

In a 250 mL flask with a magnetic bar, 100 mg of **CTF-Phen** and 50 mg of CuI were added. Then, three cycles of vacuum and nitrogen were done, and 40 mL of acetonitrile was added. The mixture was stirred for 24 h at 75 °C. The brown solid was isolated by centrifugation and repeatedly washed with acetonitrile several times. Finally, it was dried at 40 °C for several hours.

### 2.2 General Procedure for the Copper-Catalyzed Reaction

In a 19 mL vial with a magnetic bar was prepared a mixture of iodoaniline **2** (0.100 mmol), isothiocyanate **3** (0.150 mmol), DABCO (0.200 mmol), and catalyst **Cu@Phen-CTF** (10 mg, 4.6 mol %) in toluene (2 mL). The vial was sealed with a screw cap and stirred for 72 h at 50 °C. Finally, the mixture was filtrated, and the solvent of the reaction was removed under vacuum. The product was purified by flash column chromatography using cyclohexane/ethyl acetate as indicated in each case.

### 2.3 Catalyst Recycling

In a 19 mL vial with a magnetic bar was prepared a mixture of iodoaniline (**2a**) (0.100 mmol), phenylisothiocyanate (**3a**) (0.150 mmol), DABCO (0.200 mmol), and catalyst **Cu@Phen-CTF** (10 mg, 4.6 mol %) in toluene (2 mL). The vial was sealed with a screw cap and stirred for 72 h at 50 °C. Then, the reaction crude is centrifuged and washed with toluene and acetone three times each. The catalyst is recovered as a browned powder and was dried under vacuum for 2 h before the next catalytic run.

### 2.4 Leaching Experiment

For the leaching experiment, an initial catalytic run using substrate **2a** under the previously studied conditions was performed for 24 h. An aliquot was taken to check the conversion, which was calculated by ^1^H NMR (60%). Then, the crude was filtered to eliminate **Cu@Phen-CTF** and left the initial mixture in another vial at 50 °C for an additional 14 h. No evolution toward final product was observed; just the intermediate product was detected. The relationship between signals of the final product and the intermediate and the starting material is the same before and after filtration.

## 3 Results and Discussion

### 3.1 Synthesis and Characterization of the Material

To synthesize the pristine material **CTF-Phen**, we initially performed the synthesis of the symmetric building block 4,4′-(1,10-phenanthroline-3,8-diyl)­dibenzonitrile (**1**) through a double Suzuki cross-coupling between 3,8-dibromophenanthroline and 4-cyanophenylboronic acid in the presence of potassium carbonate as a base and Pd­(PPh_3_)_4_ as a catalyst at 100 °C during 72 h.^58^ Once the monomer **1** was prepared, we proceeded to the synthesis of **CTF-Phen** under the optimized solvothermal conditions described previously in the literature by the cyclization of monomer **1** in the presence of trifluoromethanesulfonic acid (TFMSA) at 100 °C (Scheme 1).^58^ The material was obtained as a powdery yellow solid. As previously reported, **CTF-Phen** material obtained through this procedure lacks crystallinity (see below).

**Scheme 1:**
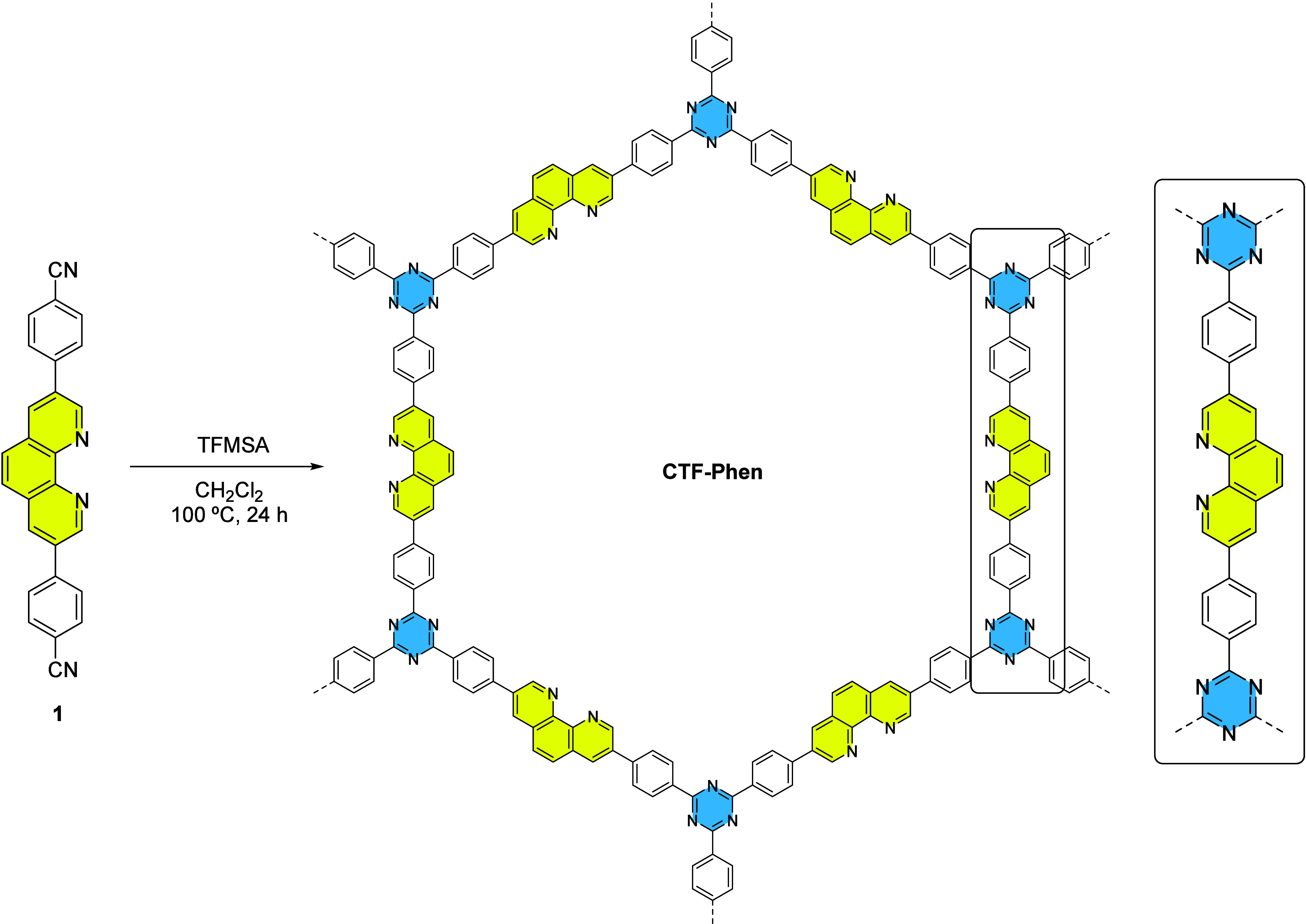
Synthesis of **CTF-Phen**

In order to functionalize the material with Cu­(I), a dispersion of 100 mg of **CTF-Phen** in acetonitrile was treated with 50 mg of CuI at 75 °C for 24 h, achieving a brown solid (Figure 2a). Interestingly, a content of 13.6 wt % of Cu, measured by TXRF,^59^ was achieved (see Supporting Information, Figure S18), which means that almost the 50% of the phenanthroline moieties present in **Cu@Phen-CTF** are coordinating Cu­(I) centers. This material was comprehensively characterized using the same set of techniques applied to pristine **CTF-Phen**, with the aim of confirming the preservation of both its microstructural features and chemical identity (bottom of Figure 2).

**Figure 2:**
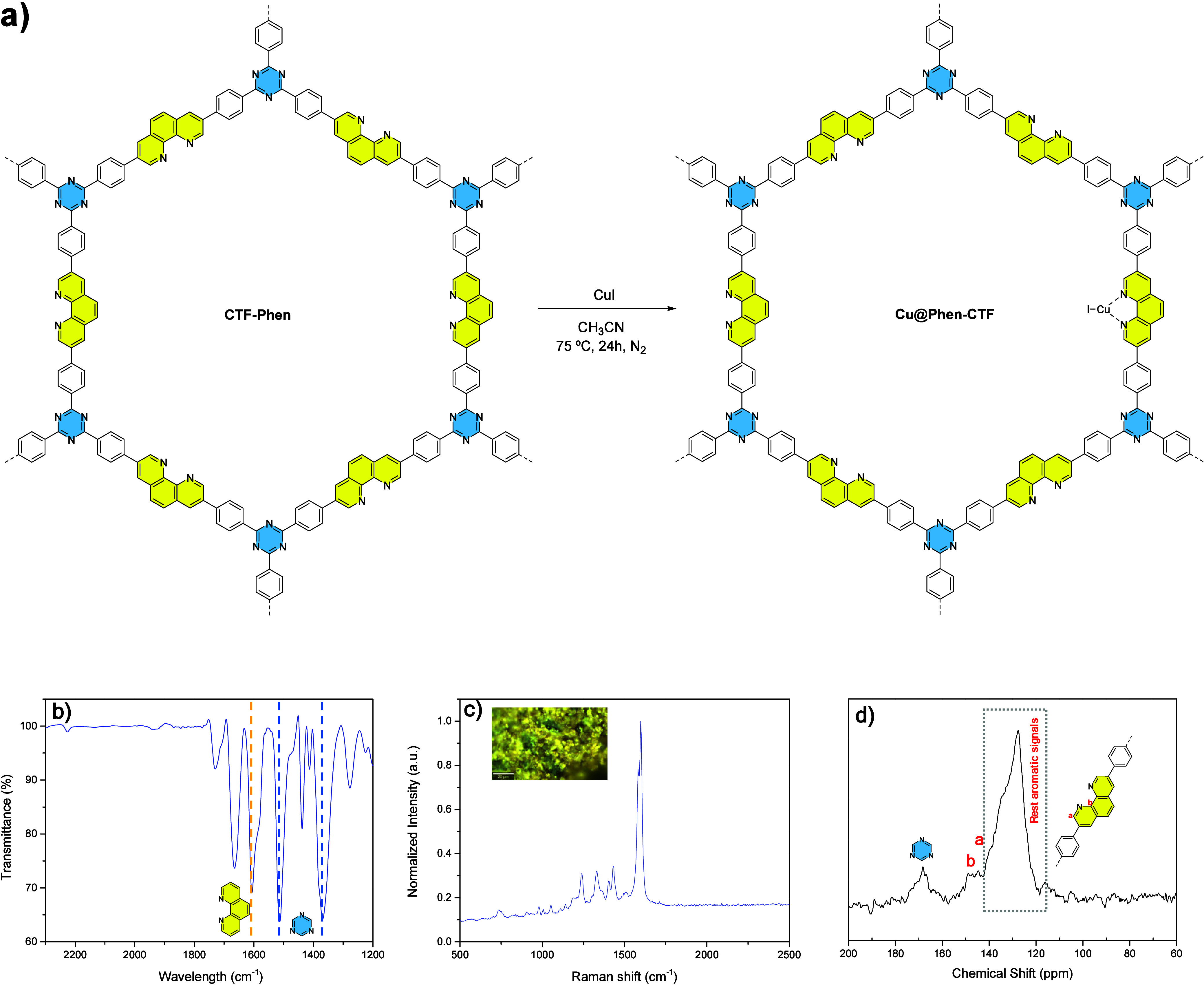
Synthesis and characterization of **Cu@Phen-CTF**. (a) Reaction conditions for the synthesis of **Cu@Phen-CTF**, (b) FTIR spectrum, (c) Raman spectrum, (d) ^13^C CP/MAS NMR spectrum.

The initial characterization of **Cu@Phen-CTF** was performed by using Fourier-transform infrared (FTIR) spectroscopy. The formation of the targeted triazine-based framework was supported by two key observations (Figure 2b): first, the near-complete disappearance of the characteristic nitrile stretching band at 2229 cm^–1^ and second, the appearance of two intense absorption bands at 1513 and 1368 cm^–1^, which are attributed to aromatic C–N stretching vibrations and the breathing modes of the triazine rings, respectively.^60^ The Raman spectra of **Cu@Phen-CTF** (Figure 2c) also showed the absence of the band associated with the nitrile group (vibration band that can be observed in the Raman spectrum of the **1-CuI complex**, see Figure S6 in the Supporting Information) as well as the presence of two main bands at 1598 and 1582 cm^–1^ that can be assigned to the presence of benzene, phenanthroline, and triazine rings.^61,62^ The formation of triazine units was further supported by solid-state ^13^C NMR spectroscopy acquired via cross-polarization magic angle spinning (CP-MAS). The spectrum displays a characteristic peak centered at approximately 168 ppm, which is attributed to the aromatic carbons of the C_3_N_3_ triazine moiety (Figure 2d).^60,63^ The remaining signals are consistent with other aromatic carbons within the framework, as evidenced by comparison with the spectra of **Cu@Phen-CTF**, **CTF-Phen**, and compound **1** reported previously in the literature.^39,40^

Thermogravimetric analysis (TGA) showed that **Cu@Phen-CTF** possesses good thermal stability, up to almost 300 °C even under an oxidant atmosphere (Figures S19 and S20 in the Supporting Information). The initial weight loss observed below approximately 150 °C can be attributed to the removal of a physically adsorbed solvent or moisture within the porous framework. At higher temperatures, the weight loss corresponds to the progressive decomposition of the organic framework. Regarding crystallinity, powder X-ray diffraction of **Cu@Phen-CTF** did not show any diffraction peaks, confirming the amorphous nature of the triazine polymer (Figure S7 in the Supporting Information). In addition, its specific Brunauer–Emmett–Teller (BET) surface area and gas adsorption measurement, was evaluated from N_2_ adsorption-desorption measurement at 77 K, afforded a negligible value of 21 m^2^/g (Figure S21 in the Supporting Information) and a lower value than obtained for the pristine material (358 m^2^/g),^58^ which suggests that the coordination of the CuI has been achieved, provoking a reduction in the porous size^64^ or the porous blockage.^65^ On the other hand, the morphology of the material was analyzed by means of scanning electron microscopy (SEM) (Figure 3a), showing an amorphous appearance with a random particle size. In conjunction with SEM analysis, energy-dispersive X-ray (EDX) analysis showed a uniform distribution of Cu and I (Figure 3c,d). In addition, the high-resolution transmission electron microscopy (TEM) (Figure 3e,f) shows the laminated structure of the **Cu@Phen-CTF** as well as the absence of Cu nanoparticles, which could catalyzed reactions.^66–68^

**Figure 3:**
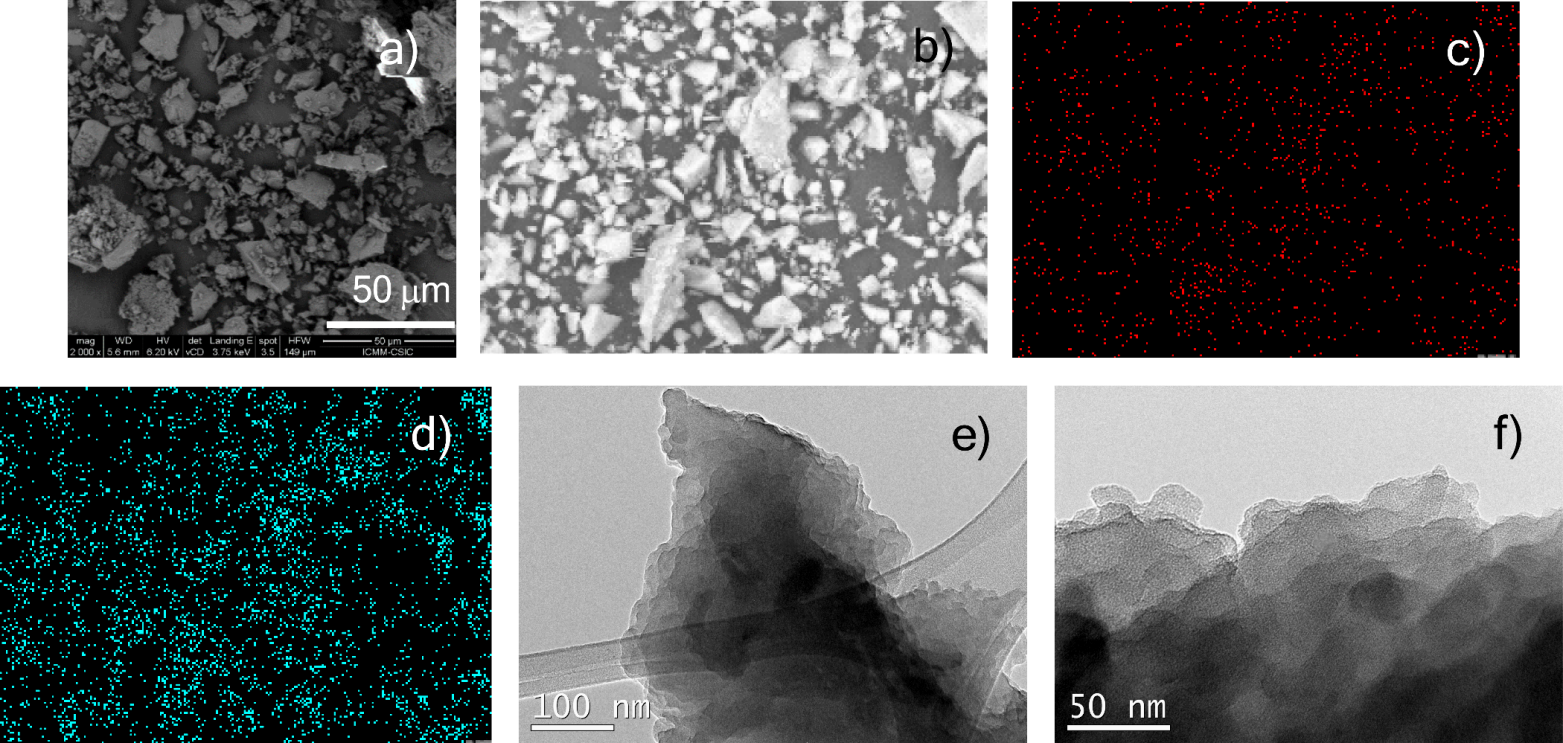
(a) SEM image, (b) SEM image for EDX analysis, (c) copper mapping, (d) iodine mapping, and (e, f) TEM images of **Cu@Phen-CTF**.

To corroborate the desired incorporation of CuI into the CTF and its coordination with phenanthroline units, we performed X-ray photoelectron spectroscopy (XPS) analysis of **Phen-CTF** and **Cu@Phen-CTF**. Indeed, the survey spectra for both materials (Figure S9 in the Supporting Information) presented the characteristic XPS C 1s (285 eV of binding energy (BE)) and N 1s (around 399 eV of BE) core-level regions. **Cu@Phen-CTF** presented additional peaks corresponding to Cu 2p (932 eV of BE) and I 2p (619 eV of BE) core-level regions because of the incorporation of CuI. Therefore, this survey analysis cross-checked that the CuI has been introduced in the structure of the material **Cu@Phen-CTF**. Analyzing the components (Figure 4), the fit of the XPS C 1s core-level region afforded two components for both materials, which may be assigned as a mixture contribution of C sp^2^ and C sp^3^ at lower BE (pink peak) and C bonded to N at higher BE (purple peak), respectively. This last peak may also be due to the presence of residual nitrile group, as could be observed in the FTIR.

**Figure 4:**
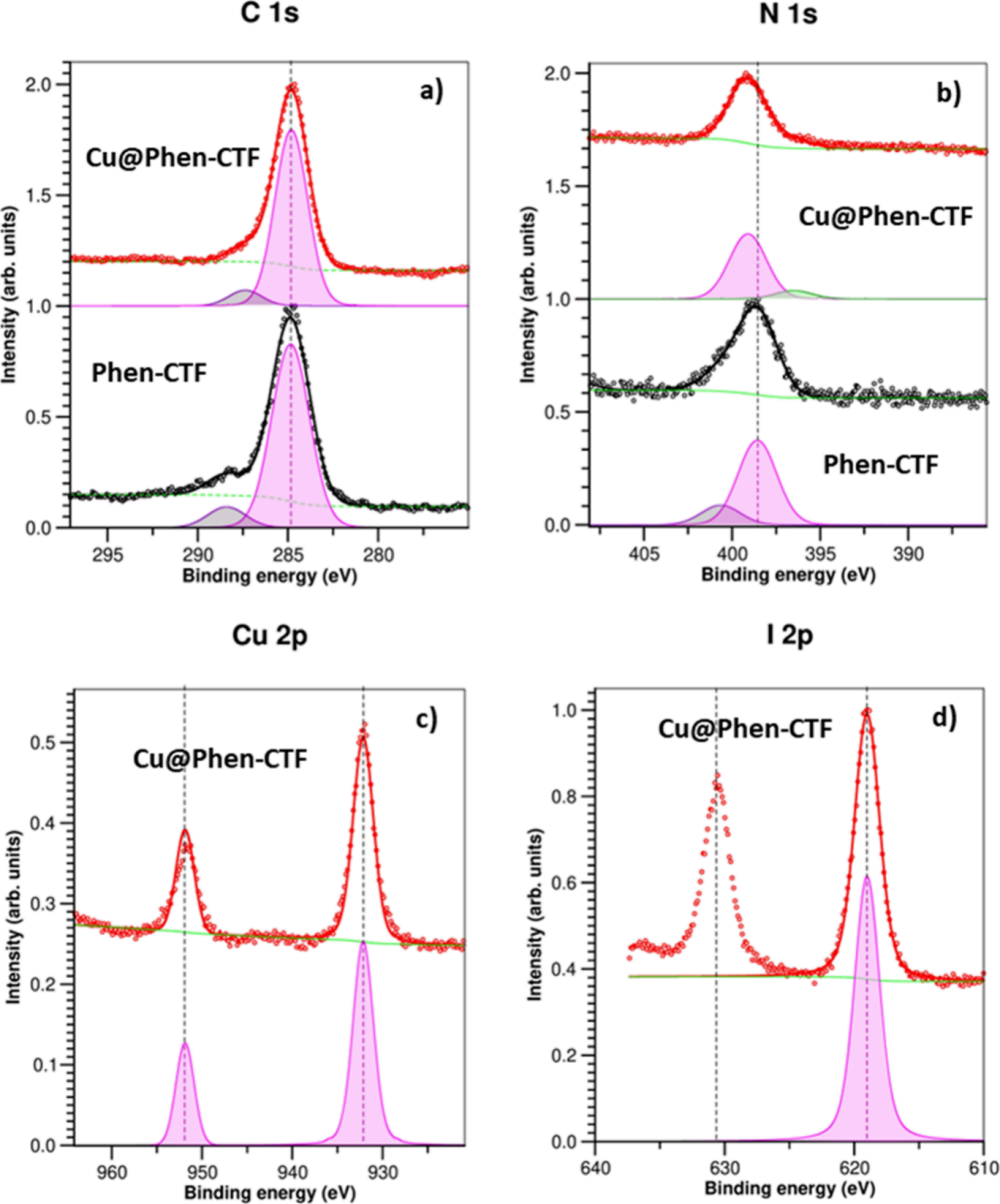
X-ray Photoelectron Spectroscopy spectra: (a) C 1s and (b) N 1s for **Cu@Phen-CTF** (top) and **Phen-CTF** (bottom) and (c) Cu 2p and (d) I 3d for **Cu@Phen-CTF**.

Regarding the fit of the N 1s core-level region for **Cu@Phen-CTF**, this shows a main peak at 399.1 eV of BE corresponding to the C=N bond. This same main peak at 398.5 eV of BE is also present in the fit of the N 1s core-level region for **Phen-CTF**; however, it is 0.6 eV more shifting than the corresponding peak for **Cu@Phen-CTF**. This fact indicated that the coordination of Cu to phenanthroline units has taken place.^69–72^ In addition, the binding energy found for Cu 3d_3/2_ in **Cu@Phen-CTF** was 931.9 eV, a value about 0.3 eV lower than the corresponding to free CuI (932.2 eV).^71,73,74^ This negative shift is indicative of coordination of Cu­(I) to the imine functionalities of **Phen-CTF**. Electron donation from the imine groups to the metal center leads to a decrease in the electron deficiency of the Cu­(I) species. Moreover, no signals were found for any other Cu species, such as Cu­(II) and metallic Cu. The same shifting effect was observed for iodine atom, whose BE values were 619.0 eV and 0.7 eV lower than the corresponding value for CuI salt.^71^ These findings support the effective immobilization of Cu­(I) and point to a strong metal–ligand interaction between the phenanthroline moieties and the copper centers.

### 3.2 Catalytic Application

Once the material was prepared and characterized, we checked its applicability as a heterogeneous metal catalyst. For that reason, we selected the catalytic tandem reaction of 2-iodobenzenamines **2a** with isothiocyanate **3a** to synthesize 2-aminobenzothiazoles **4a**. This reaction runs efficiently using CuI under mild conditions and in the presence of 1,10-phenanthroline as the best ligand.^23^ Initially, we utilized the best reaction conditions described by Wu et al., which consisted of the use of CuI and DABCO (as acid trapping) in toluene at 50 °C. In our case, we replaced the molecular CuI by the heterogeneous catalyst **Cu@Phen-CTF** (entry 1, Table 1) and achieved the 2-aminobenzothiazole **4a** in a 93% isolated yield after 72 h. This result is very impressive because it is similar to that obtained in homogeneous conditions (entries 7 and 8, Table 1) although after major reaction time (generally, reaction times are higher under heterogeneous conditions than under homogeneous ones). Then, we checked if variations of initial conditions could afford similar results, but in any case, they were not reached (entries 2–4, Table 1). Only, when the reaction was carried out at room temperature, a lower yield (79%) was achieved.

**Table 1:**
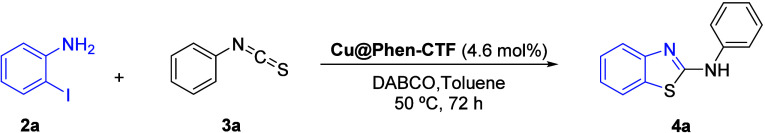
Screening of Conditions and Control Experiments for the Synthesis of 2-Aminobenzothiazole **4a** ^a^

entry	variations from conditions	yield^b^
1	none	93%
2	r.t. instead of 50 °C	79%
3	no DABCO	
4	no material	
5	24 h	60% (62%)^c^
6	**Phen-CTF** as catalyst	
7	**1**-CuI complex (10 mol %) as catalyst	98%^d^
8	CuI as catalyst (10 mol %)	88%^e^

a Reactions were performed using 0.100 mmol of **2**, 0.150 mmol of **3**, 0.200 mmol of DABCO, 10 mg (4.6 mol %) of **Cu@Phen-CTF**, and 2 mL of toluene.

b Isolated yield.

c Conversion into parentheses.

d Reaction was performed using 0.100 mmol of **2**, 0.150 mmol of **3**, 0.200 mmol of DABCO, 0.01 mmol (10 mol %) of CuI, and 0.02 mmol of monomer **1** during 23 h.

e Data from ref 9.

On the other hand, the decreasing reaction time, from 72 to 24 h, provoked a lower conversion (62%) (entry 5, Table 1). Finally, to determine if the pristine material **Phen-CTF** was enabled to run the reaction, we carried out the reaction with it under the same reaction conditions that in the case of the **Cu@Phen-CTF**, obtaining null conversion (entry 6, Table 1). This result showed that the presence of Cu­(I) is indispensable to the reaction take place. Considering the results obtained, we selected the reaction conditions of entry 1 in Table 1 to be used in the reaction scope (Scheme 2). It should be noted that despite the amorphous nature of the **Cu@Phen-CTF** material, as confirmed by powder X-ray diffraction (PXRD), its lack of crystallinity did not negatively affect its catalytic performance. This can be attributed to the homogeneous distribution of copper active sites coordinated to phenanthroline units throughout the polymeric network, as evidenced by SEM-EDX analysis (Figure 3c). Therefore, the high efficiency observed in C–S bond formation, along with the excellent recyclability, suggests that crystallinity is not a critical requirement for catalytic activity in this system. In order to evaluate the scope of the reaction, the tandem reaction between different 2-iodobenzenamines **2** with isothiocyanate **3a** using **Cu@Phen-CTF** as a catalyst was first studied. As shown in Scheme 2, our system allowed us to carry out the reaction in the presence of different electron-withdrawing and electron-donating groups in different positions of the 2-iodoaniline **2** (substrates **4b**–**4g**, **4h**–**4l**, and **4m**). In most cases, the presence of substituents at position 4 or 5 of **2** reached good to high yields independently of the functional group nature. Only when a trifluoromethyl group is present at C-4 or C-5 of **2** (**2d** or **2l**), the corresponding 2-aminobenzothiazole **4d** or **4l** was obtained in lower yield (63% and 56%, respectively). In addition, the substitution at position 4 for a halogen atom (**2f** or **2g**) also afforded lower yields (53% and 54%, respectively). This fact can be explained by the lower nucleophilicity of the nitrogen atom by the presence of an electron-withdrawing group because the first step in this process is the nucleophilic attack of aniline to the isothiocyanate. Finally, the reaction with 2-chloro-6-iodoaniline (**2m**) produced the final compound **4m** in low yield (27%).

**Scheme 2:**
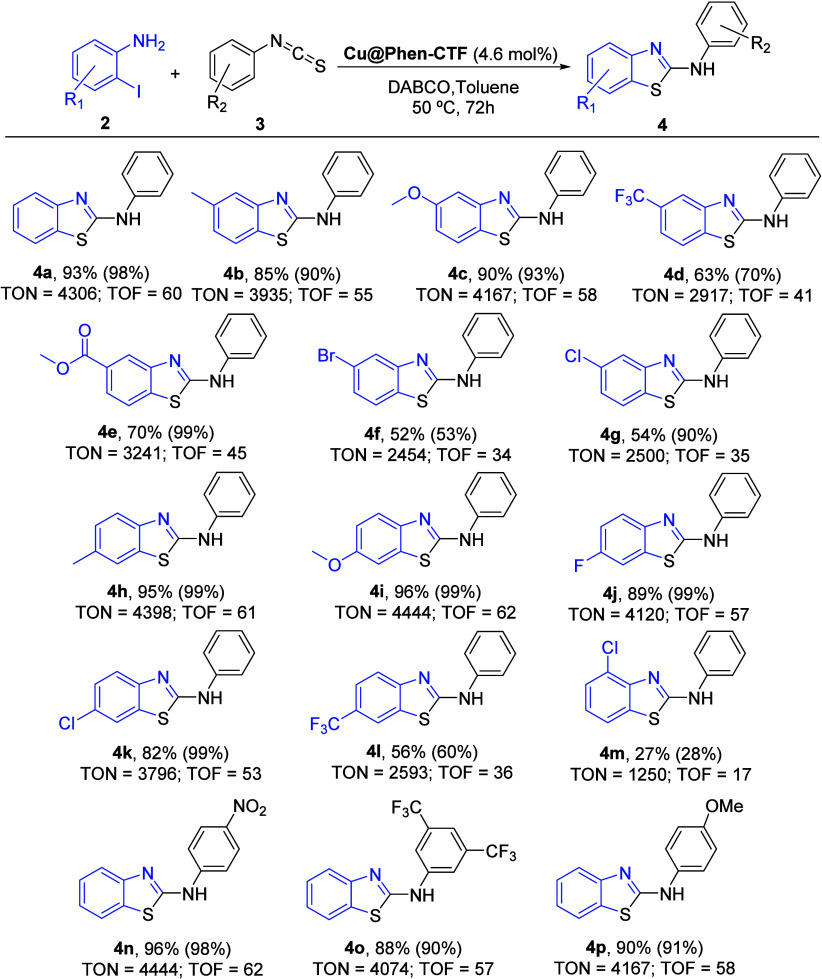
Scope Reaction^a^

a The reactions were carried out using 0.100 mmol of **2**, 0.150 mmol of **3**, 0.200 mmol of DABCO, and 10 mg of **Cu@Phen-CTF** in 2 mL of toluene at 50 °C during 72 h. The yields, TON, and TOF (h^–1^) were calculated from the isolated product. Reaction conversion into parentheses.

Finally, to determine the reaction scope regarding the isothiocyanate, several of them were used. In all cases, the tandem reaction worked well independently of the functional group present and their electronic nature, obtaining the final product **4n**–**p** in high yield (88–96%). The TON observed in almost all cases were very high (4400–3500) regardless of the substituent present except for the derivatives with chloride, bromide, trifluoromethyl, and ester atoms where the values are a bit lower (3000 and 2500). Only, for compound **4m**, which bears a chlorine atom at position 2, the achieved TON dropped drastically.

Once the catalytic capacity of our material was checked, we carried out several analyses to demonstrate its structural and chemical integrity. First, it should be noted that, after catalysis, neither was observed a change in the coloration to blue indicative of the oxidation to Cu­(II). The morphology and uniform distribution of Cu and I throughout the material, determined by SEM (Figure 5a) and EDX analyses (Figure 5b–d and Figure S16 in the Supporting Information), was maintained after their use in the catalyzed reactions. Moreover, the TEM images indicated that no Cu clusters were formed during the coupling reactions (Figure 5e,f). Likewise, the XPS analysis of **Cu@Phen-CTF** does not show the core-level region for Cu­(II) (Figure S10 in the Supporting Information), which exposed the great stability conferred by the coordination to the phenanthroline units present in the material that avoid their oxidation. Also, the Cu/I, Cu/N, and N/I ratios before and after catalysis remain constant, which indicates that (i) no leaching of CuI had taken place, (ii) the very stable DABCO-CuI complex has been not formed,^75^ and (iii) iodine that could be generate does not stay in the pores as has been described for other triazine-based materials.^76,77^ Furthermore, ICP-OES analysis of the reaction solution (see Table S1 in the Supporting Information), obtained by filtration of the reaction crude, indicated that the degree of Cu­(I) leached into the reaction media is very small. In addition, the Raman and FTIR analyses of the material before and after reaction are identical (compare Figures S2–S5 in the Supporting Information), bringing to light the great stability of the **Cu@Phen-CTF** (despite the Raman of the pristine material could not be recorded due to their high fluorescence, the fact that **Cu@Phen-CTF** did not show this process is an indirect result that indicates the integrity of the **Cu@Phen-CTF**).

**Figure 5:**
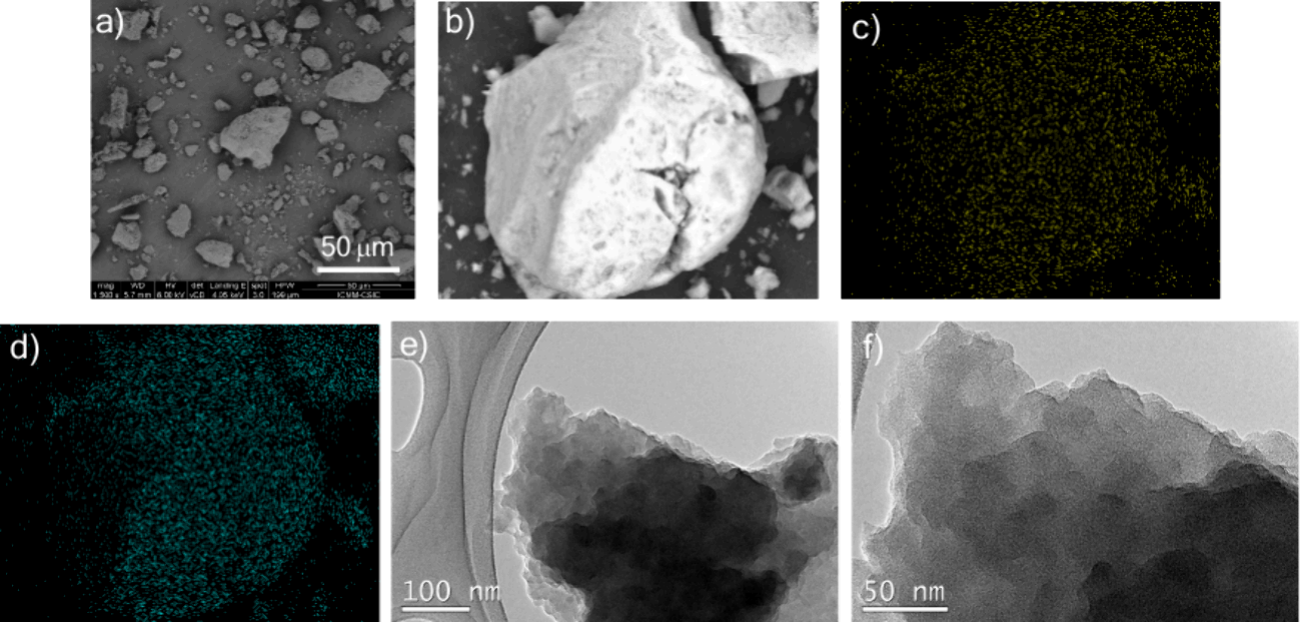
(a) SEM image, (b) SEM image for EDX analysis, (c) copper mapping, (d) iodine mapping, and (e, f) TEM images of **Cu@Phen-CTF** after catalysis.

### 3.3 Recyclability and Leaching Tests

A key requirement for heterogeneous functionalized materials is the suppression of leaching of active molecular species into the reaction medium.^78^ This aspect is critical, as one of the main advantages of heterogeneous catalysis lies in the straightforward recovery of the catalyst—typically by simple filtration—while minimizing the transfer of undesired species into the isolated product fractions.

To evaluate potential metal leaching, a catalytic run using substrate **2a** was conducted under previously optimized conditions for 24 h. An aliquot of the reaction mixture was taken and analyzed by ^1^H NMR, showing 60% conversion (see Figure S24 in the Supporting Information). The reaction mixture was then filtered to remove the **Cu@Phen-CTF** catalyst, and the filtrate was transferred to a new vial and stirred at 50 °C for an additional 14 h (see Figure S25 in the Supporting Information). No further conversion to the final product was observed, with only the intermediate remaining and a small amount of **2a**, indicating that no active copper species remained in solution. This fact indicates that no catalytic species were leached into the reaction media, which agree with the results obtained by XPS, TXRF, and ICP-OES analyses commented on before.

In addition, the recyclability and sturdiness of **Cu@Phen-CTF** were assessed by performing consecutive reactions under identical conditions and under 24 or 72 h reaction time. After each run, the catalyst was recovered by filtration, washed, and reused directly. The **Cu@Phen-CTF** catalyst maintained high activity over almost six consecutive cycles, with only a slight decrease in the yield observed (Figure 6). In addition, we have included a benchmark comparison with other copper-based heterogeneous materials (see Table S2, Supporting Information), showing that this material delivers comparable or significantly superior results in terms of conversion and recyclability.

**Figure 6:**
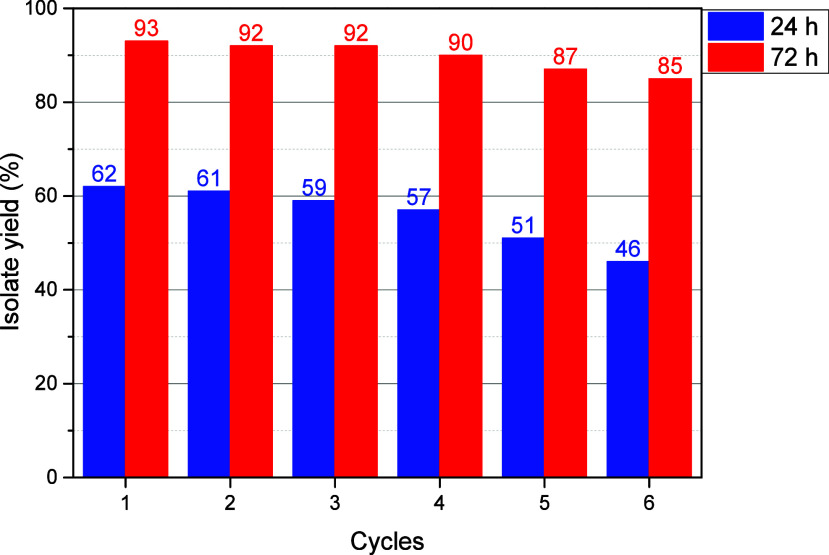
Recycling of **Cu@Phen-CTF**. In the blue barrel, the reaction was performed at 24 h, and in the red barrel, it was at 72 h.

### 3.4 Mechanism Proposal

Based on the experimental results and previous reports on copper-catalyzed syntheses of benzothiazoles,^79^ a plausible catalytic cycle for the formation of 2-aminobenzothiazoles using **Cu@Phen-CTF** is proposed (Scheme 3). The catalytic process begins with the formation of the active Cu­(I) species immobilized within the phenanthroline sites of the covalent triazine framework. The strong chelating interaction between Cu­(I) and the phenanthroline units stabilizes the metal center and generates the catalytically active Cu­(I)-phenanthroline complex within the porous polymeric network (see above for leaching studies and stability). In parallel, the *o*-iodoaniline reacts with the isothiocyanate under basic conditions to form the corresponding thiourea intermediate. Deprotonation of this intermediate by DABCO generates a sulfur-centered nucleophilic species that can interact with the Cu­(I) center. Subsequently, oxidative addition of the aryl iodide to the Cu­(I) complex affords a higher-valent Cu­(III) intermediate. Finally, reductive elimination releases the 2-aminobenzothiazole product and regenerates the Cu­(I) catalytic species embedded within the CTF framework, thus closing the catalytic cycle. The immobilization of the Cu­(I) centers within the phenanthroline-functionalized polymeric structure likely contributes to the high stability of the catalyst and prevents metal leaching during the reaction.

**Scheme 3:**
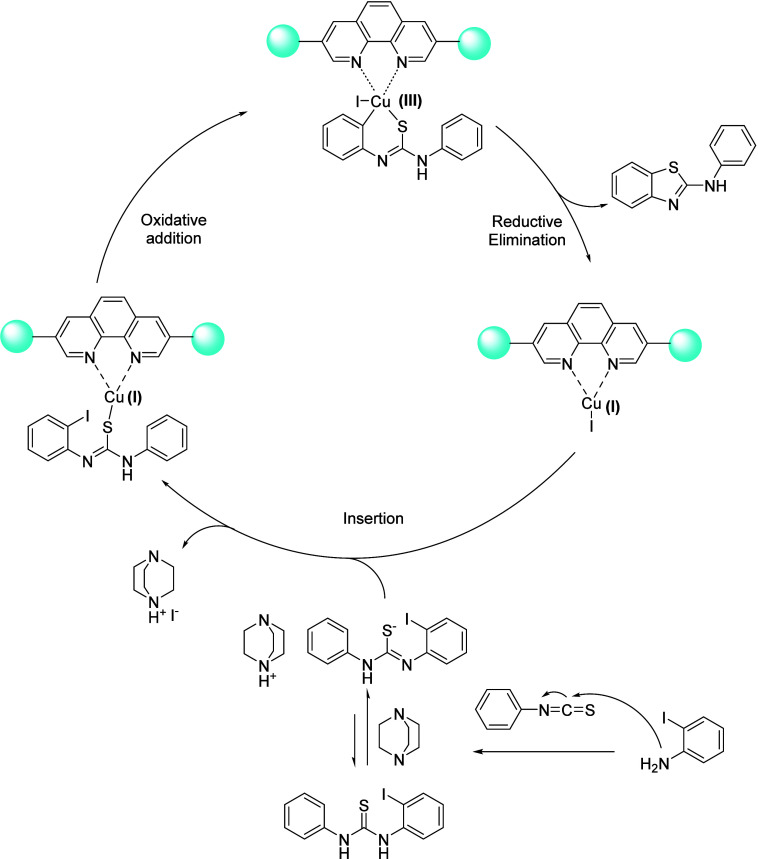
Mechanism Proposal for the Formation of the 2-Aminobenzothiazoles

## 4 Conclusions

A new triazine-based covalent triazine framework (CTF) functionalized with 1,10-phenanthroline units was successfully synthesized. These units serve as coordination sites for Cu­(I), introduced via postsynthetic metalation. The resulting catalyst, **Cu@Phen-CTF**, was thoroughly characterized by using FTIR, Raman, ^13^C solid-state NMR, SEM/EDX, TEM, TXRF, XPS, and BET, confirming successful Cu­(I) incorporation and structural stability. The **Cu@Phen-CTF** catalyst demonstrated excellent activity in the tandem cyclization of 2-iodoanilines with isothiocyanates, enabling the efficient formation of 2-aminobenzothiazoles, which are pharmacologically relevant scaffolds. The reaction proceeds under mild conditions (50 °C, toluene, 72 h) and tolerates a broad range of functional groups, affording high to excellent yields. Compared with traditional homogeneous Cu­(I)/phenanthroline catalysts, this heterogeneous system offers the significant advantage of recyclability. It can be easily recovered and reused across multiple catalytic cycles with a minimal loss of performance. Leaching tests confirmed that no active copper species were released into the reaction medium, indicating a true heterogeneous catalysis. XPS analysis revealed strong coordination between Cu­(I) and the phenanthroline ligands, which stabilizes the metal and prevents its deactivation and oxidation. This work provides a robust and sustainable catalytic platform for C–S bond formation, expanding the utility of phenanthroline-based CTFs in the synthesis of bioactive molecules and contributing to the development of a green and efficient heterogeneous catalyst.

## Supplementary Material



## Notes

Material and general methods, experimental procedures, characterizations of materials (including additional figures), and NMR spectra of aminobenzothiazoles (PDF)
