# Supplementary material for: Design and Application of a Phenanthroline-Based Covalent Triazine Framework with Cu(I) for the Heterogeneous Synthesis of 2‑Aminobenzothiazoles
Source: ACS Appl Mater Interfaces. 2026 May 4;18(19):27682–93. doi: 10.1021/acsami.6c05338 (PMC13195569; doi:10.1021/acsami.6c05338)
Supplement: Supplementary file 1 [file am6c05338_si_001.pdf]

Supporting Information

Design and Application of a Phenanthroline-Based Covalent Triazine Framework with Cu(I) for the Heterogeneous Synthesis of 2-Aminobenzothiazoles

Jorge Vega,[a] Maria Jose Capitán,[b,c] Jesus Alvarez Alonso,[b,d,e,f] Alberto Fraile,[a,g,*] José Alemán[a,g,*]

[a] *Organic Chemistry Department, Science Faculty, Universidad Autónoma de Madrid, 28049 Madrid, Spain.*

[b] *Departamento de Física de la Materia Condensada, Universidad Autónoma de Madrid, 29049-Madrid, Spain*

[c] *Física de sistemas crecidos con baja dimensionalidad, Universidad Autónoma de Madrid, Unidad Asociada al CSIC por el IEM, DP, 29049-Madrid, Spain*

.[d] *Instituto de Ciencia de Materiales "Nicolás Cabrera", Univ. Autónoma de Madrid, 28049-Madrid, Spain*

[e] *Instituto de Física de la Materia Condensada IFIMAC, Univ. Autónoma de Madrid, 28049-Madrid, Spain*

[f] *Instituto de Estructura de la Materia IEM-CSIC, 28006-Madrid, Spain*

[g] *Institute for Advanced Research in Chemical Sciences (IAdChem), Universidad Autónoma de Madrid, 28049 Madrid, Spain.*

Corresponding Authors: Alberto Fraile (E-mail: alberto.fraile@uam.es), José Alemán (E-mail: jose.aleman@uam.es)

Table of Contents

1. **Materials and general methods** S3
2. **Characterization of Cu@Phen-CTF** S6
   2.1. **^{13}C NMR solid-state spectroscopy** S6
   2.2. **Infrared spectroscopy** S6
   2.3. **RAMAN spectroscopy** S7
   2.4. **Powder X-ray diffraction** S9
   2.5. **X-Ray Photoelectron Spectroscopy spectra** S10
   2.6. **SEM images and mapping** S11
   2.7. **EDX analyses** S12
   2.8. **TXRF analyses** S13
   2.9. **Thermogravimetric analysis** S14
   2.10. **N_2 adsorption-desorption isotherm** S15
3. **Recyclability experiment** S16
4. **Leaching experiment** S17
5. **Synthesis and ^{1}H NMR data of 2-aminobenzothiazoles** S21
6. **NMR Spectra** S26
7. **References** S35

1. Materials and general methods

All reagents and solvents were purchased from commercial sources and used without further purification.

Nuclear Magnetic Resonance (NMR) spectra were acquired on a *Bruker AV-300 spectrometer*, running at 300 MHz for ^{1}H. Chemical shifts (δ) are reported in ppm relative to residual solvent signals ($CDCl_3$: 7.26 ppm for ^{1}H NMR). Data for ^{1}H NMR are reported as follows: chemical shift (δ ppm), multiplicity (brs = broad singlet, s = singlet, d = doublet, t = triplet, m = multiplet), coupling constant (Hz) and integration. Solid-state ^{13}C (100.61 MHz) CPMAS NMR spectra have been obtained on a *Bruker AV-400 WB spectrometer* at 300 K using a 4mm triple channel probe head (BL4 X/Y/^{1}H). Samples were carefully packed in a 4-mm diameter cylindrical zirconia rotor with Kel-F end-caps. Operating conditions involved 2.75 μs 90° ^{1}H pulses and decoupling field strength of 90.9 kHz by TPPM sequence. The rotor spin rate was set at 10 kHz. Relaxation delay of 4 s and a contact time of 3 ms. ^{13}C spectra were originally referenced to an adamantane sample and then the chemical shifts were recalculated to the Me_4Si [for the CH_2 atom δ(adamantane)=29.5 ppm].

IR spectra were recorded in a *Perkin-Elmer 283* equipped with *ATR MIRacle Single Reflection Horizontal*.

Raman spectra were collected using a WITEC-ALPHA300R using a laser with an excitation wavelength of 532 nm (0.5 mW), focused on the sample with a 100x objective. The presented spectra were the mean over all the data acquired.

Thermogravimetric analyses (TGA) tests were performed on a *TA Instruments Q500* thermobalance equipped with an EGA furnace. Pt and N_2 sample holders were used as purge gas with a flow rate of 90 mL min^{-1}. The method used is a heating ramp of 10 °C/min from room temperature up to 1000 °C.

Powder X-ray difraction was obtained in a *X'Pert PRO* difractometer θ/2θ geometry from *Panalytica*l equipped with a *Johansson* monochromator for ʎ K_{α}, a *X'Celerator* fast detector in an alumina holder. The θ/2θ swept was performed from 4 to 45º with an angular increase of 0.0167º/100 s. Simulated model structures of PXRD were carried out using Materials Studio 8.0 Program.

Scanning Electron Microscopy (SEM) images were carried out on a *Hitachi S-3000N electron microscope* with a coupled ESED detector and an analyzer from energy dispersive X-ray from *Oxford Instruments*, *INCAx-sight* model. The images were

obtained in vacuum after being metallized in a *Sputter Quórum Q150T-S* with gold coating.

Transmission Electron Microscopy (TEM) images were acquired with a JEOL-JEM 2100F instrument equipped with a CCD high resolution camera and an Oxford EDX spectrometer in situ microprobe. Samples were drop casted from 0.5 mg mL^{-1} nanotube methanol suspensions on holey-carbon copper grids.

X Ray Photoelectron Spectroscopy (XPS) have all been carried out under ultra-high vacuum (UHV) conditions. The experimental chamber has a base pressure of 2×10^{-10} mbar and is equipped with an X-ray source with a Mg anode whose Kα emission line produces photons of energy hv = 1253.6 eV which are used for X-ray photoemission spectroscopy (XPS) measurements. A He discharge lamp provides He-I (hv = 21.2 eV) and He-II (hv = 40.8 eV) photons for ultraviolet photoemission spectroscopy (UPS). For both techniques a hemispherical energy analyzer (LEYBOLD LHS10) has been used. The pass energy of the analyzer was set to 50 eV for the XPS measurements to reach a resolution of 0.7 eV and 5 eV for the UPS measurements reaching a final resolution of 0.1 eV. Sample preparation consisted of glueing the powders using an Ag liquid colloid to avoid samples falling in the UHV chamber and sample charging. All the core levels are referred to the Ag 3d5/2 XPS core level (BE = 367.4 eV). The energies of the UPS spectra are referred also to the Fermi edge of the Ar^{+}-ion sputtered Au(111) crystal. The measured spectra have been deconvoluted using a Richardson–Lucy algorithm in order to eliminate the Mg Kα intrinsic line width and satellites.[1,2] An iterative Richardson–Lucy procedure was applied until convergence, using as stopping criteria the appearance of a maximum in the Shannon entropy. The core level peaks, have been fitted, after subtraction of a Tougaard[3] background with a Doniach-Sunjic combination of Lorentzian and Gaussian lineshapes.[4] The measured XPS intensity was corrected by the corresponding atomic sensitive factor of each edge. Thus, the corrected intensity is proportional to the average atomic composition of the films following standard procedures and using the atomic sensitivities determined previously for this spectrometer type.[5,6] Assuming that the films are strictly homogeneous within the escape depth of the electrons, the ratio of the intensities of two atoms core level peaks is related to the atomic density ratio (XA/XB) by XA/XB = A IA /IB where A = 1/SA / 1/SB and SA and SB are the atomic sensitive factor determined for the pure chemical elements for the specific electron analyzer used.[7]

Total X-Ray Fluorescence analyses (TXRF): Qualitative and quantitative were performed with a benchtop S2 PicoFox TXRF spectrometer from Bruker Nano (Germany) 1,2. TXRF system was equipped with a Mo X-ray source working at 50 kV

and 600 μA, a multilayer monochromator with 80% of reflectivity at 17.5 keV (Mo Kα), a XFlash SDD detector with an effective area of 30 mm^2 and an energy resolution better than 150 eV for 5.9 keV (Mn Kα). For deconvolution and integration commercial Spectra v. 7.5.3 software package from Bruker was used. Previously to the measurements, sample acid digestions were performed in a high pressure and temperature microwave. Acid digestion technology was used by mean of an UltraWAVE digestion system from Millestone (Italy) with a single reaction chamber able of operates up to 199 bar pressure and 270 °C.

2. Characterization of Cu@Phen-CTF

2.1. ^{13}C NMR solid-state spectroscopy

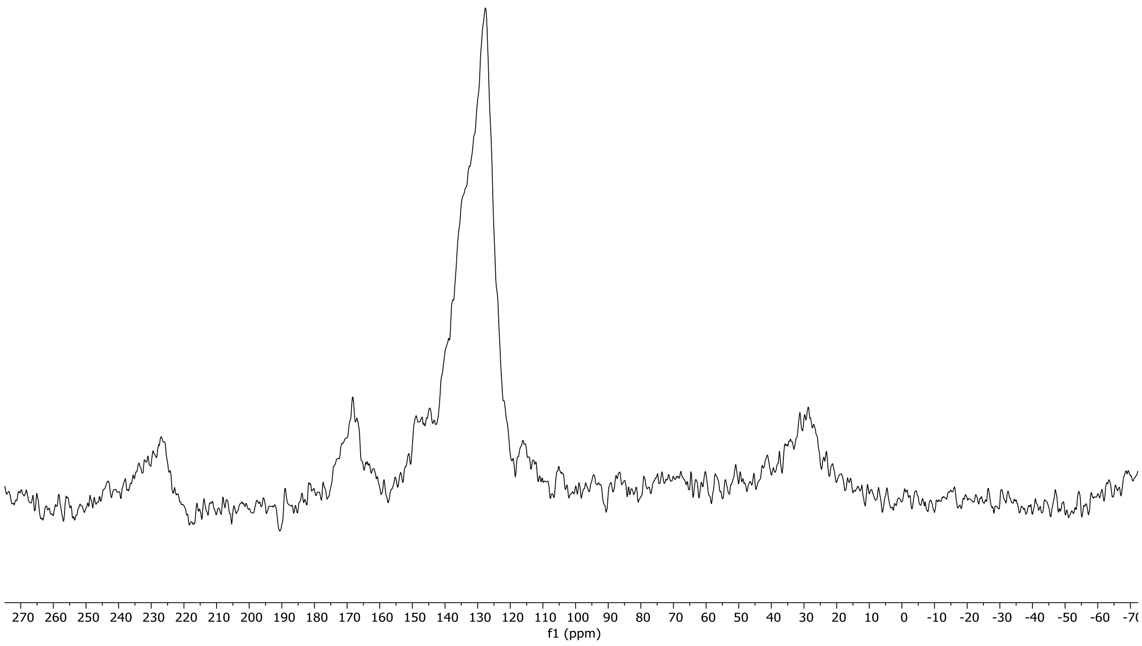

Figure S1. ^{13}C NMR-CP-MAS spectra of **Cu@Phen-CTF**.

2.2. Infrared spectroscopy

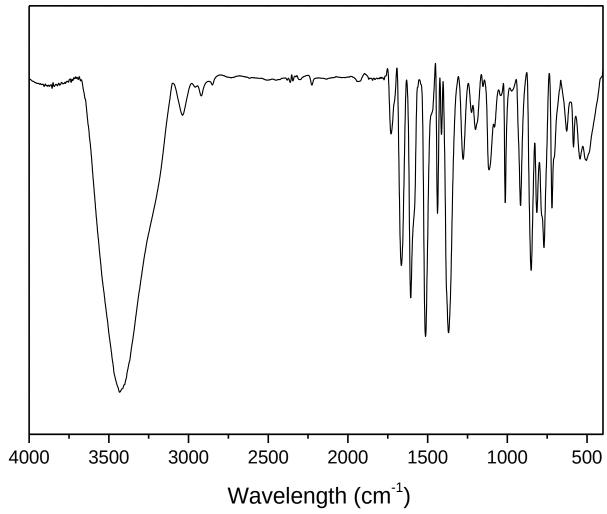

Figure S2. FTIR spectrum of **Cu@Phen-CTF**.

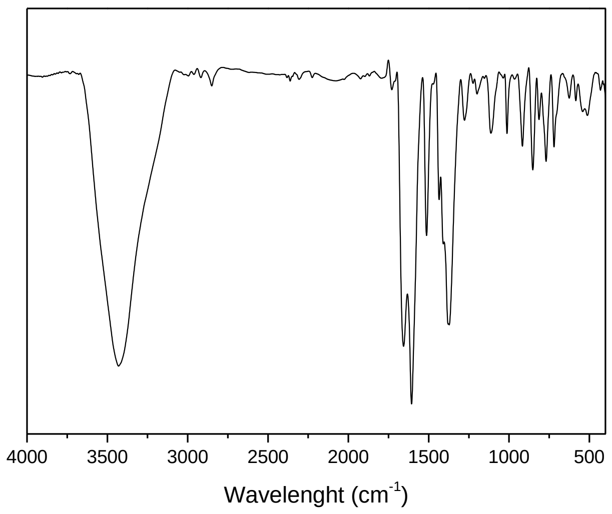

Figure S3. FTIR spectrum of **Cu@Phen-CTF** after catalysis.

2.3. RAMAN spectroscopy

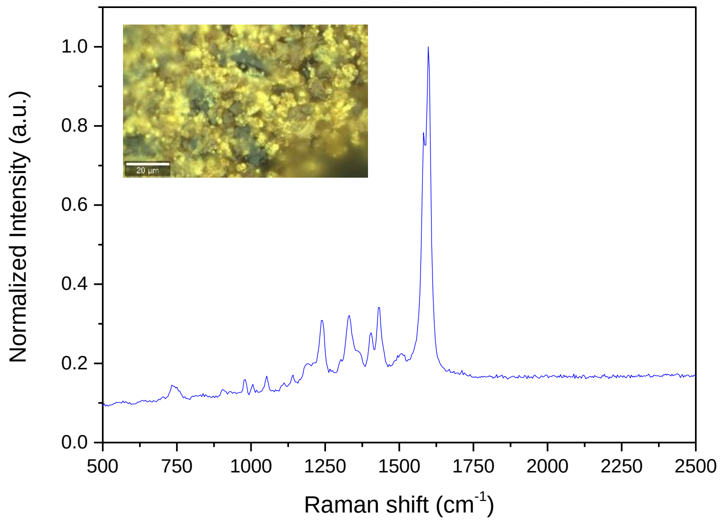

Figure S4. RAMAN spectrum of **Cu@Phen-CTF**.

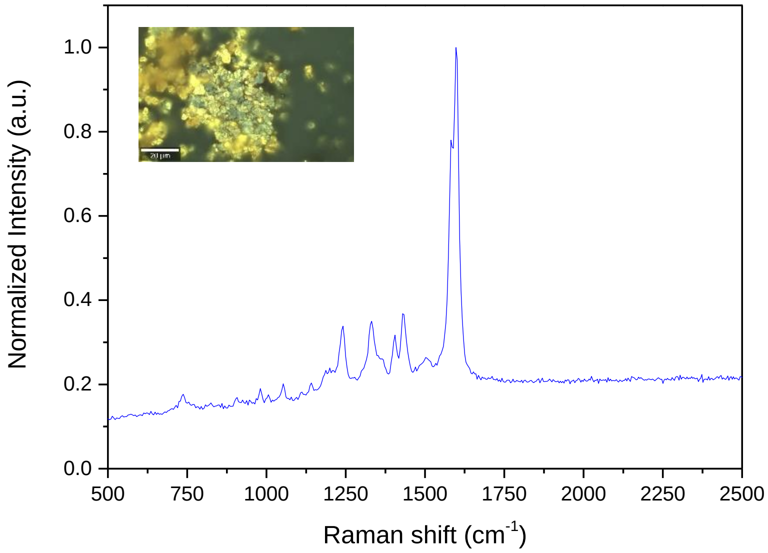

Figure S5. RAMAN spectrum of **Cu@Phen-CTF** after catalysis.

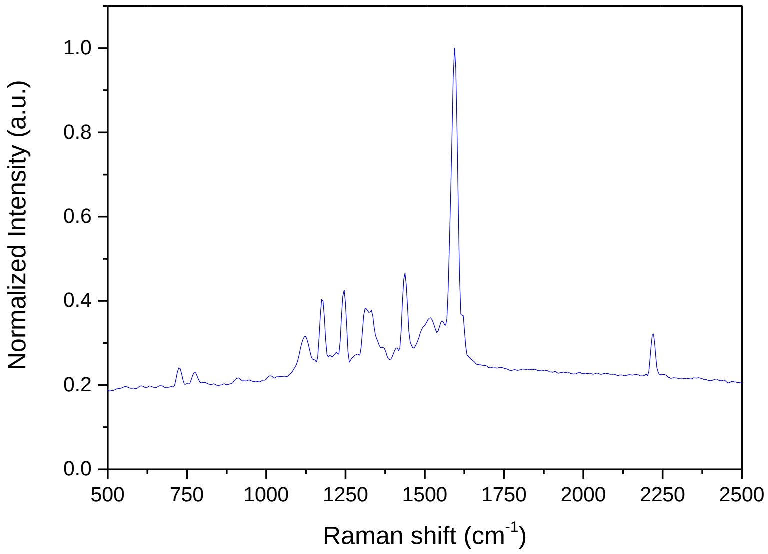

Figure S6. RAMAN spectrum of monomer **1**.

2.4. Powder X-ray diffraction

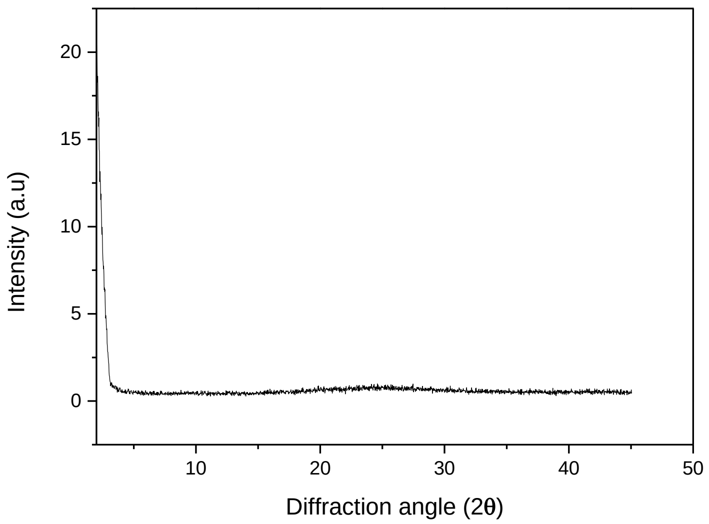

Figure S7. PXRD spectra of **Cu@Phen-CTF** before catalysis.

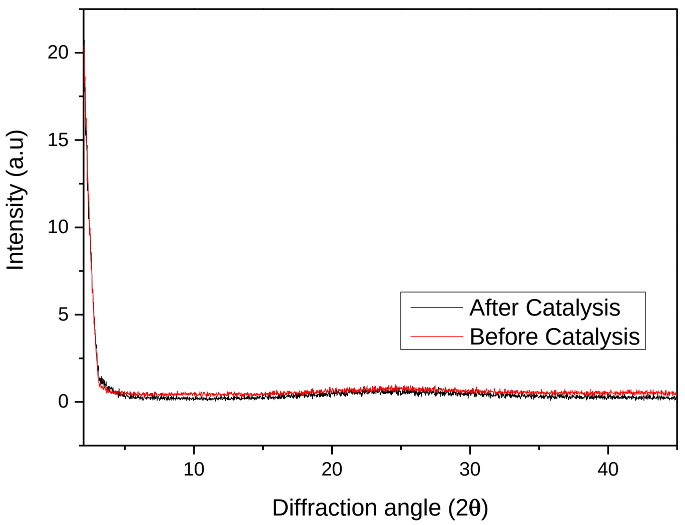

Figure S8. Comparation of PXRD spectra of **Cu@Phen-CTF** before and after catalysis.

2.5. X-Ray Photoelectron Spectroscopy spectra

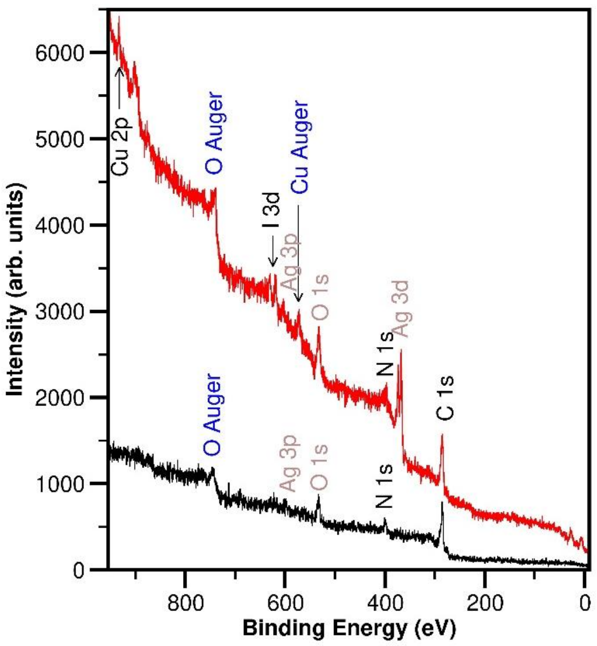

Figure S9. Survey XPS: red line for **Cu@Phen-CTF** and black line for **Phen-CTF**.

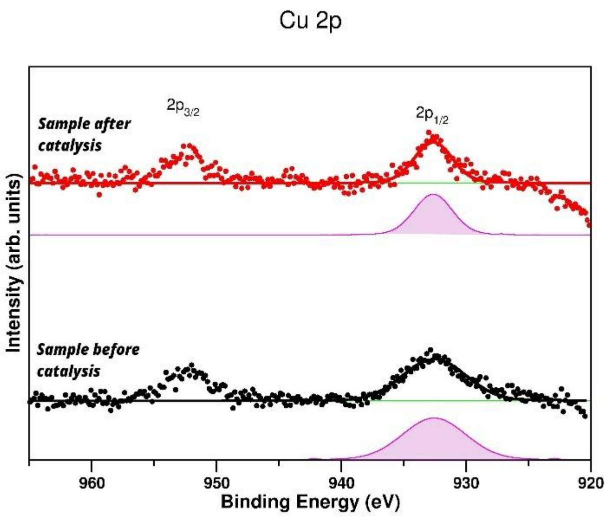

Figure S10. X-Ray Photoelectron Spectroscopy spectra of Cu 2p for **Cu@Phen-CTF** before and after catalysis.

2.6. SEM images and mapping

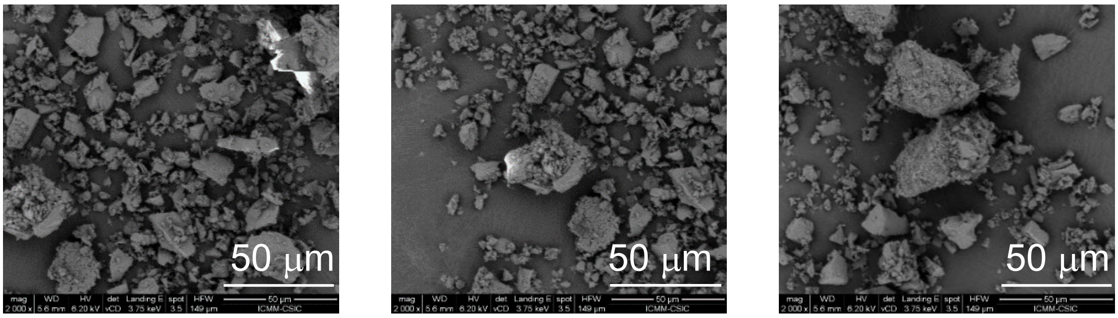

Figure S11. Several SEM images for **Cu@Phen-CTF** before catalysis.

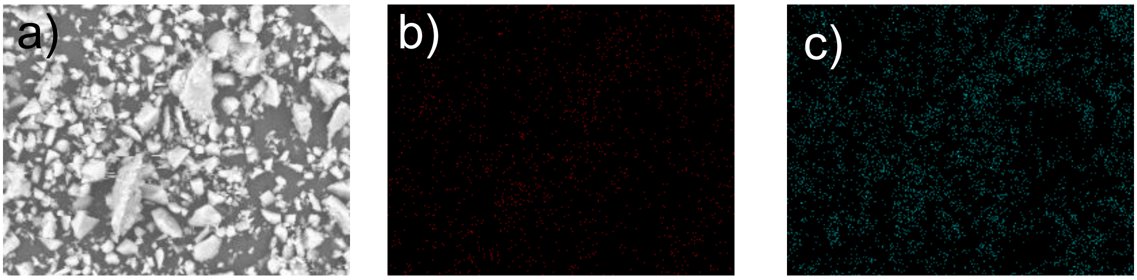

Figure S12. a) SEM image for EDX, b) Copper mapping, c) Iodine mapping for **Cu@Phen-CTF** before catalysis.

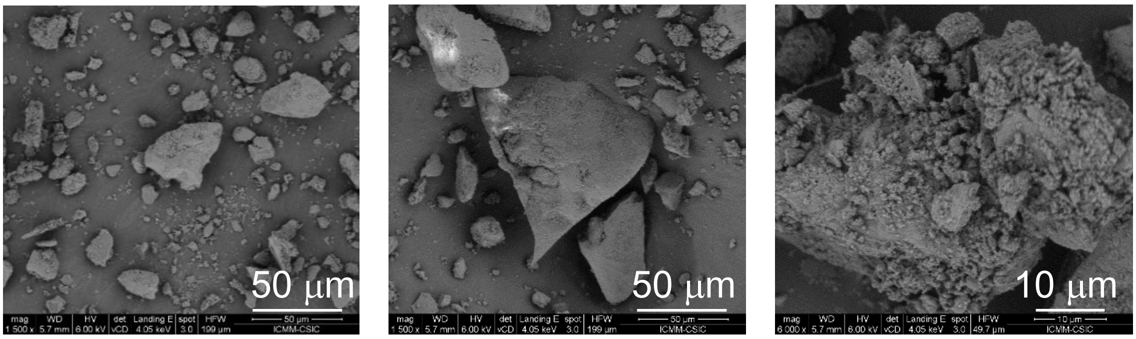

Figure S13. Several SEM images for **Cu@Phen-CTF** after catalysis.

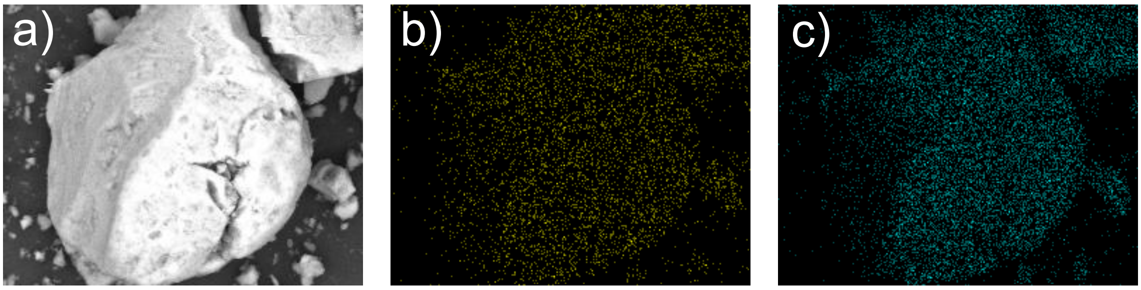

Figure S14. a) SEM image for EDX, b) Copper mapping, c) Iodine mapping for **Cu@Phen-CTF** after catalysis.

2.7. EDX analyses

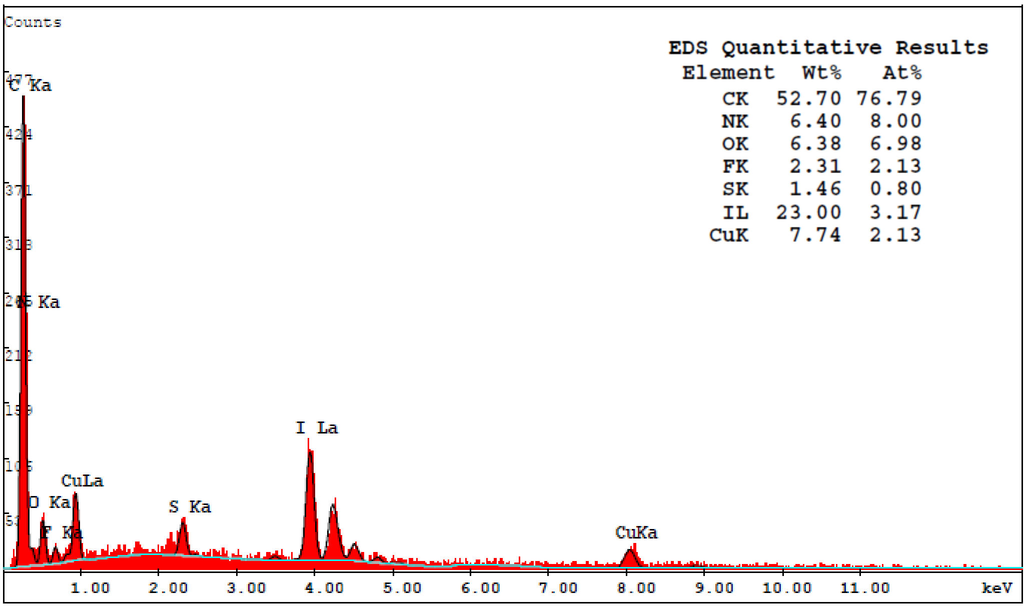

Figure S15. EDX for **Cu@Phen-CTF** before catalysis.

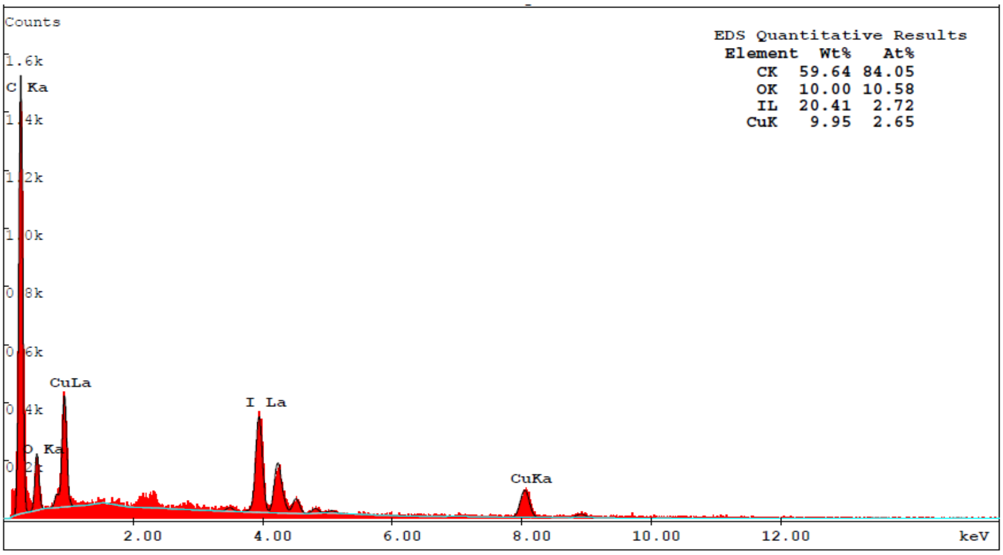

Figure S16. EDX for **Cu@Phen-CTF** after catalysis.

2.8. TXRF analyses

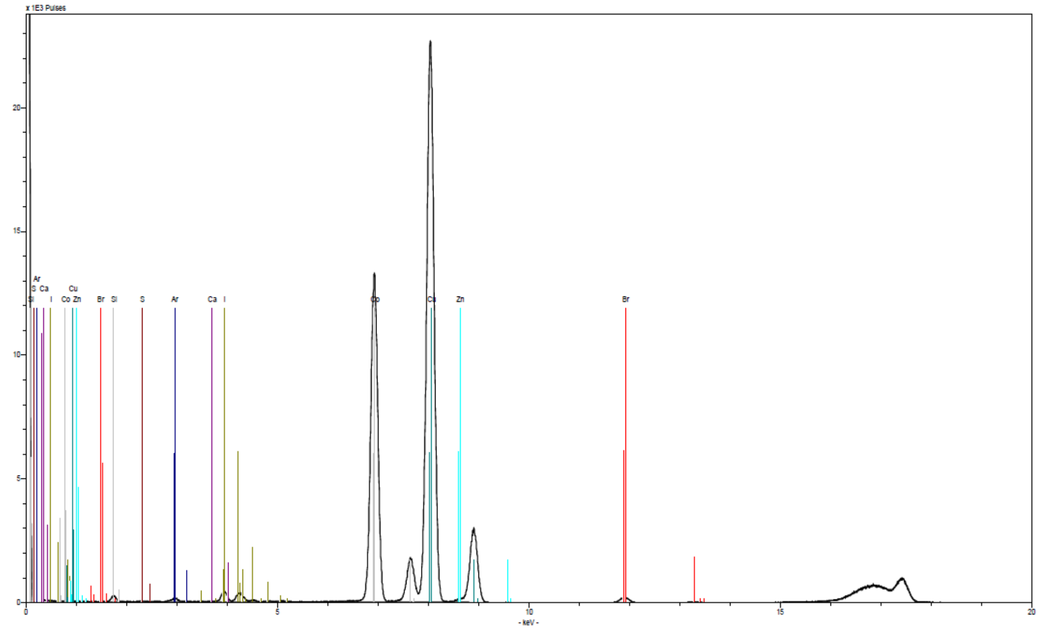

Figure S17. TXRF qualitative analyze for **Cu@Phen-CTF** before catalysis.

Element	Line	Conc./ mg/l	Sigma/ mg/l	RSD/ %	LLD/ mg/l	Net area	Backgr.	Chi
Si	K12	1059	20	1.9	25	5236	1708	1.87
S	K12	25.8	3.5	13.7	7.0	468	1773	1.22
Ca	K12	4.05	0.80	19.7	1.61	320	1803	1.05
Co (IS)	K12	1000.0	3.1	0.3	0.4	435691	3293	8.71
Cu	K12	1245.9	3.3	0.3	0.5	778548	10980	20.92
Zn	K12	3.885	0.095	2.4	0.107	2837	676	1.28
Br	K12	7.327	0.093	1.3	0.045	8494	302	0.89
I	L1	269.9	2.5	0.9	1.8	20350	2028	0.97

Figure S18. TXRF quantitative analyze for **Cu@Phen-CTF** before catalysis. Total sample concentration = 9150 mg L^{-1} (ppm).

2.9. Thermogravimetric analysis

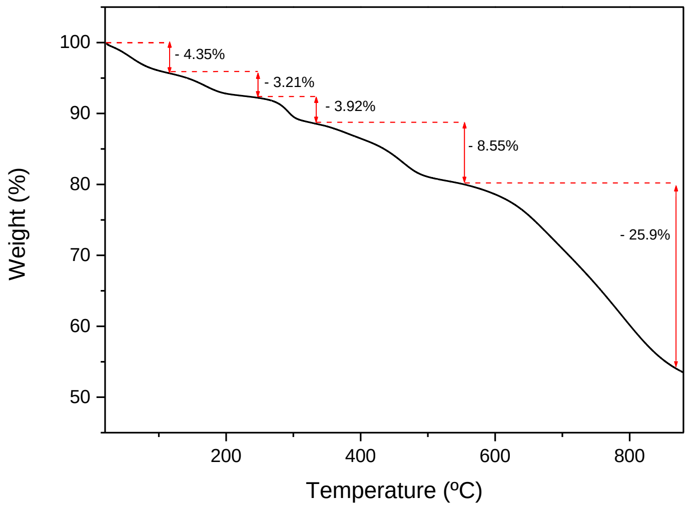

Figure S19. Thermogravimetric analysis of **Cu@Phen-CTF** heating the sample from 25 ºC to 900 ºC at 10 ºC min^{-1} under nitrogen atmosphere.

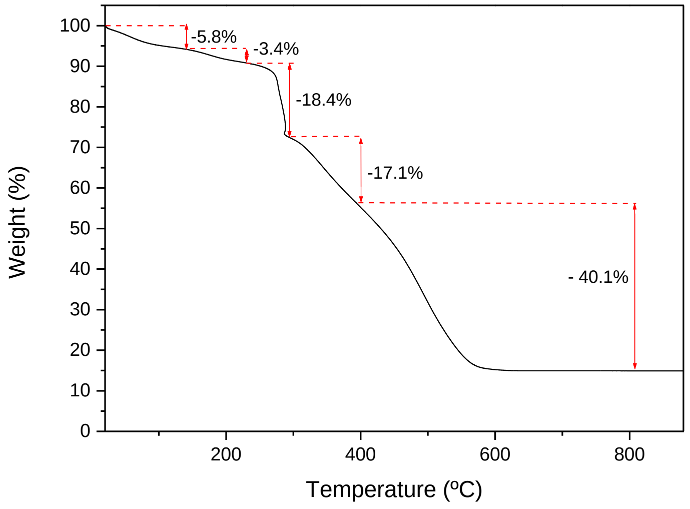

Figure S20. Thermogravimetric analysis of **Cu@Phen-CTF** heating the sample from 25 ºC to 900 ºC at 10 ºC min^{-1} under air atmosphere.

2.10. N_2 adsorption-desorption isotherm

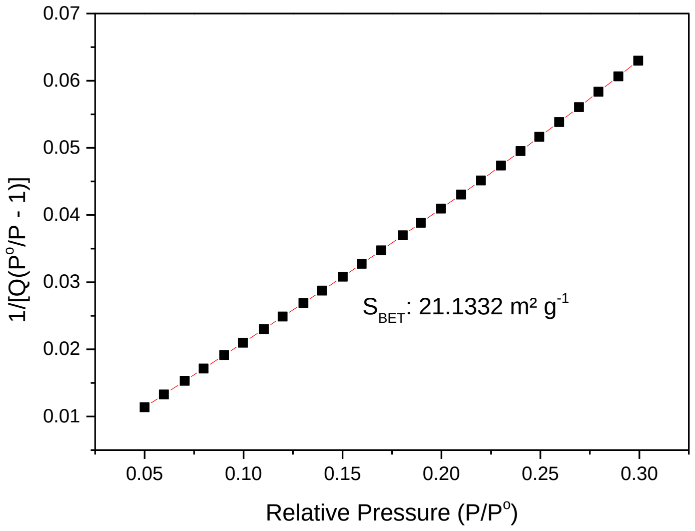

Figure S21. N_2 adsorption isotherm of **Cu@Phen-CTF**.

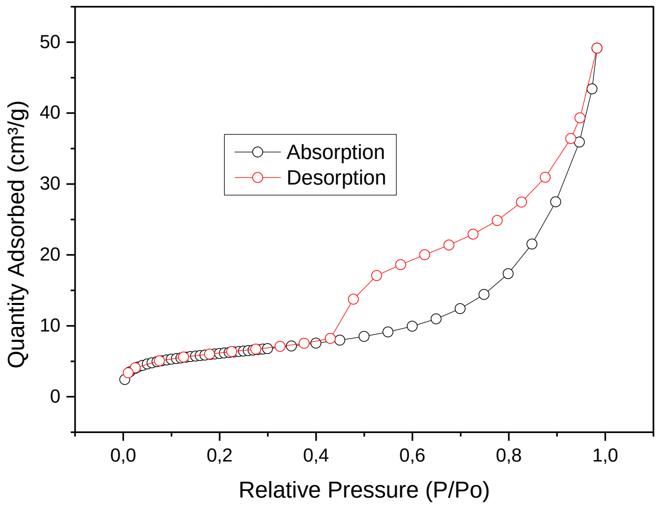

Figure S22. N_2 adsorption-desorption isotherm of **Cu@Phen-CTF**.

3. Recyclability experiment

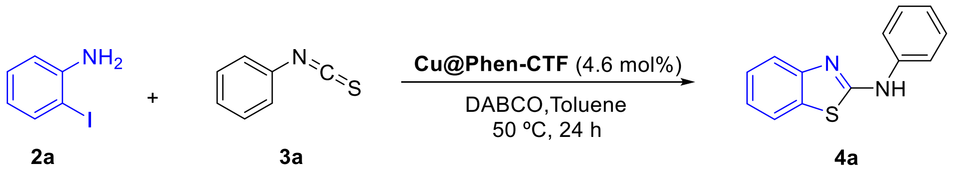

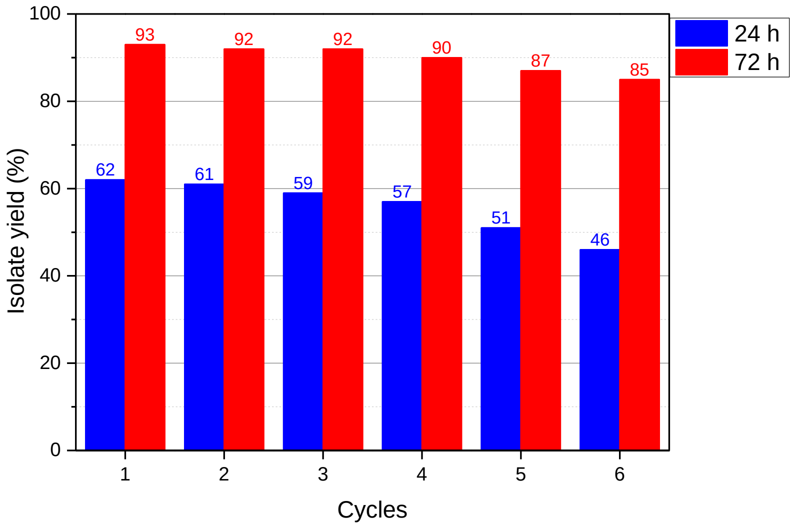

Figure S23. Recycling of **Cu@Phen-CTF.** In blue barrel the reaction was performed for 24 h and in red barrel was for 72 h.

4. Leaching experiment

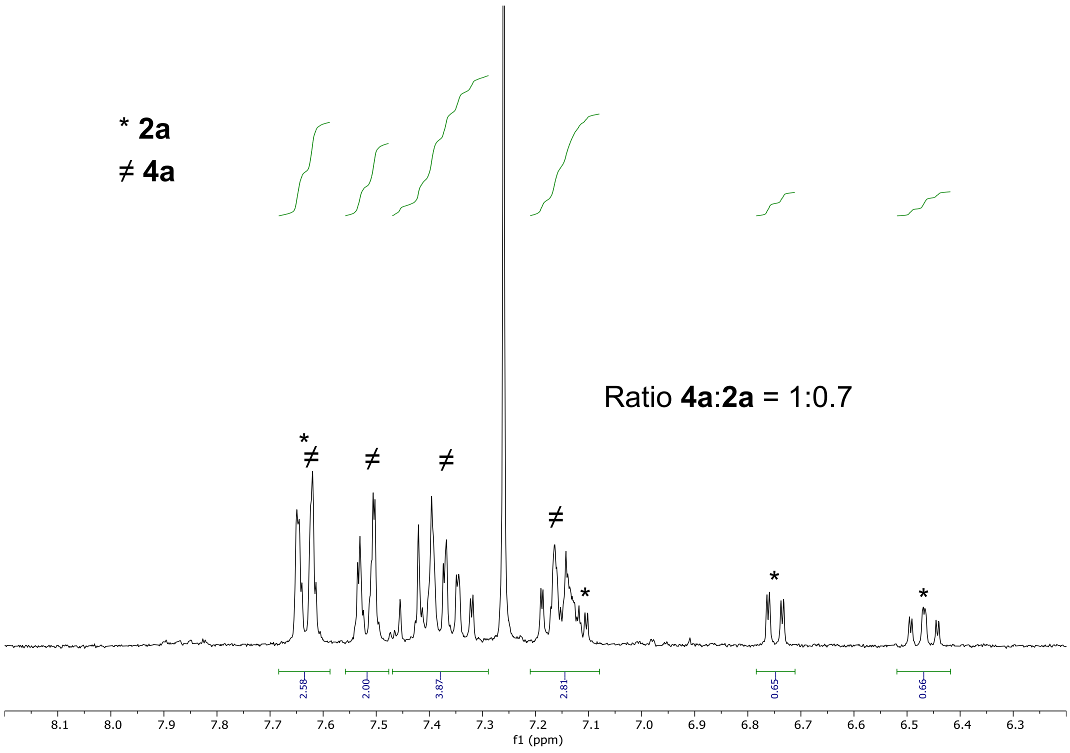

Figure S24. ^{1}H MNR of an aliquot after 24h of reaction (300 MHz, 298 K, $CDCl_3$)

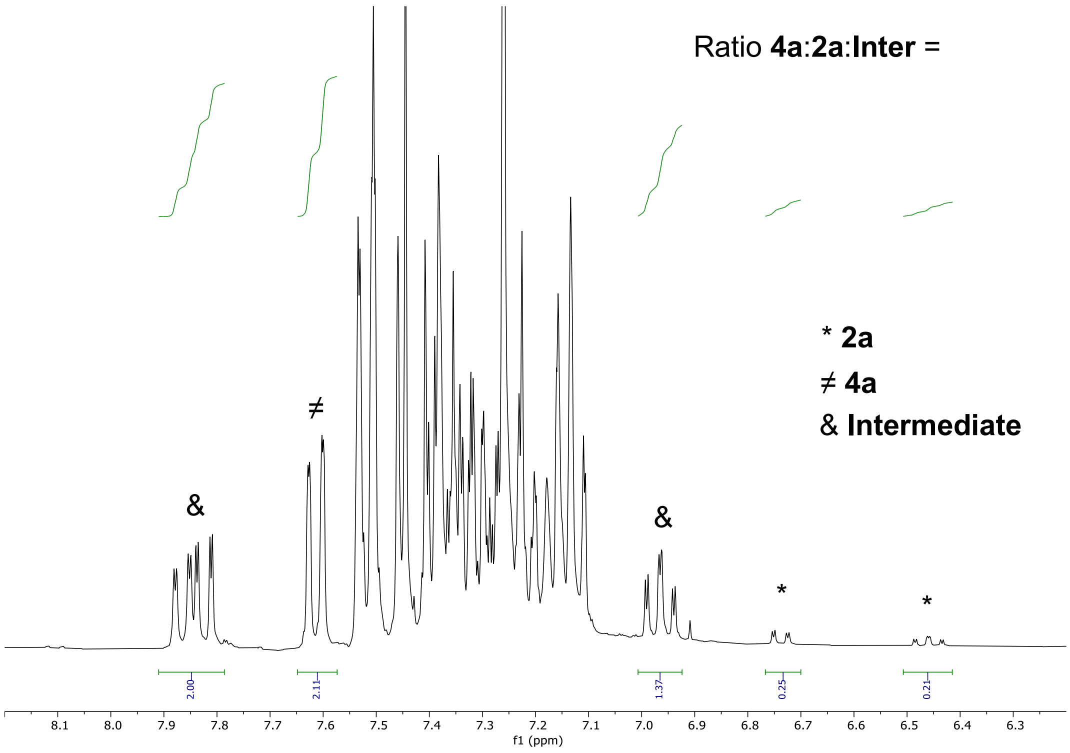

Figure S25. ^{1}H MNR of an aliquot after additional 14 h of reaction after filtration of the **Cu@Phen-CTF** (300 MHz, 298 K, $CDCl_3$).

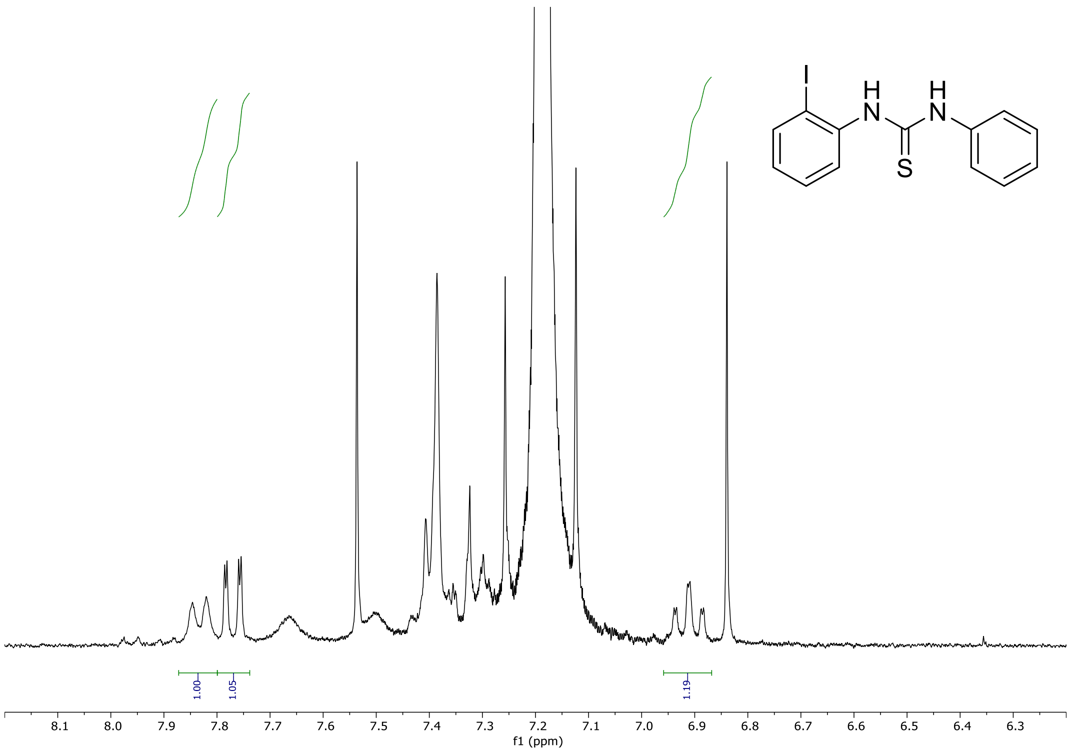

Figure S26. ^{1}H MNR of the reaction intermediate (300 MHz, 298 K, $CDCl_3$).

Table S1. ICP-OES data before and after catalysis, and reaction supernatant.

Before catalysis

Seq. No.	28	AS Loc:	2	Date:	11/14/2025 1:41:08PM
Sample ID:	JV-665_antes				
Analyte		Conc (Calib)	Conc (Sample)	Net Intensity	Corr. Intensity
Cu 327.393					
		2.286 mg/L	2.286 mg/L	266,345.8	265,927.4
		2.304 mg/L	2.304 mg/L	268,358.3	267,939.8
		2.327 mg/L	2.327 mg/L	271,072.1	270,653.7
	Mean:	2.306 mg/L	2.306 mg/L		268,173.6
	SD:	0.0209 mg/L	0.0209 mg/L		
	%RSD:	0.91			

After catalysis

Seq. No.	30	AS Loc:	4	Date:	11/14/2025 1:48:17PM
Sample ID:	JV-665_después				
Analyte		Conc (Calib)	Conc (Sample)	Net Intensity	Corr. Intensity
Cu 327.393					
		2.145 mg/L	2.145 mg/L	250,320.2	249,901.7
		2.159 mg/L	2.159 mg/L	251,911.0	251,492.5
		2.184 mg/L	2.184 mg/L	254,796.5	254,378.0
	Mean:	2.163 mg/L	2.163 mg/L		251,924.1
	SD:	0.0200 mg/L	0.0200 mg/L		
	%RSD:	0.92			

Reaction supernatant

Seq. No.	31	AS Loc:		Date:	11/14/2025 1:54:40PM
Sample ID:	JV-665_sobrenadante				
Analyte		Conc (Calib)	Conc (Sample)	Net Intensity	Corr. Intensity
Cu 327.393					
		-0.055 mg/L	-0.055 mg/L	567.9	149.4
		-0.052 mg/L	-0.052 mg/L	862.6	444.1
		-0.054 mg/L	-0.054 mg/L	668.6	250.1
	Mean:	-0.054 mg/L	-0.054 mg/L		281.2
	SD:	0.0013 mg/L	0.0013 mg/L		
	%RSD:	2.45			

Table S2. A comparison of **Cu@Phen-CTF** with some reported some reported catalysts for the cross-coupling reaction between 2-iodoaniline and phenyl isothiocyanate.

Entry	Catalyst	Conditions	Yields, (number of Cycles)	Ref
1	Merrifield resin supported phenanthroline Cu(I) complex	Tol, 60 ºC, 8 h	95 (10)	8
2	MCM-41-2N-$CuSO_4$	DMSO, 80 ºC, 8 h	95 (10)	9
3	RMOF-3-PC-CuI	Tol,60 ºC, 24 h	94 (6)	10
4	Nano Copper oxide	PEG-400, 80 ºC, 8 h	91 (3)	11
5	Fe_3O_4@EDTA–Cu(II) nanoparticles	H_2O, 50 ºC, 3 h	94 (5)	12
6	Copper(II) on Silica-Coated Magnetite Nanoparticles	H_2O, 50 ºC, 2 h	93 (5)	13
7	PhenPAF	H_2O, 80 ºC,2 h	99 (5)	14
8	Phen-COF	H_2O, 25 ºC, 28 h	99 (7)	15
9	**Cu@Phen-CTF**	Tol, 50 ºC, 72 h	93 (6)	This study

5. Synthesis and ^{1}H NMR data of 2-aminobenzothiazoles

***N*-phenylbenzo[*d*]thiazol-2-amine (4a)**

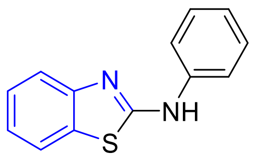

Following the general procedure, from 2-iodoaniline (**2a**) and phenyl isothiocyanate (**3a**), compound **4a** was obtained in 93% yield (21.1 mg, 0.093 mmol) as a white solid after purification by flash column chromatography (CyHx/EtOAc: 20/1 → 9/1). Spectroscopic data were consistent with the literature data for this compound.[16]

^{1}H NMR (300 MHz, $CDCl_3$) δ 9.11 (bs, 1H), 7.64 (dd, *J* = 7.8, 1.2 Hz, 1H), 7.57 (d, *J* = 8.1 Hz, 1H), 7.54 – 7.48 (m, 2H), 7.45 – 7.38 (m, 2H), 7.36 – 7.29 (m, 1H), 7.21 – 7.13 (m, 2H).

5-Methyl-*N*-phenylbenzo[*d*]thiazol-2-amine (4b)

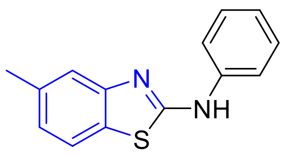

Following the general procedure, from 2-iodo-5-methylaniline (**2b**) and phenyl isothiocyanate (**3a**), compound **4b** was obtained in 85% yield (20.4 mg, 0.085 mmol) as a white solid after purification by flash column chromatography (CyHx/EtOAc: 9/1 → 4/1). Spectroscopic data were consistent with the literature data for this compound.[17]

^{1}H NMR (300 MHz, $CDCl_3$) δ 8.09 (s, 1H), 7.54 – 7.46 (m, 3H), 7.45 – 7.33 (m, 3H), 7.20 – 7.08 (m, 1H), 7.03 – 6.96 (m, 1H), 2.43 (s, 3H).

5-Methoxy-*N*-phenylbenzo[*d*]thiazol-2-amine (4c)

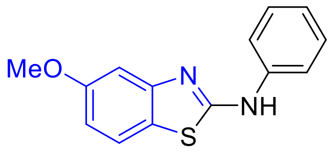

Following the general procedure, from 2-iodo-5-methoxyaniline (**2c**) and phenyl isothiocyanate (**3a**), compound **4c** was obtained in 90% yield (23.1 mg, 0.090 mmol) as a white solid after purification by flash column chromatography (CyHx/EtOAc: 20/1 → 9/1). Spectroscopic data were consistent with the literature data for this compound.[17]

^{1}H NMR (300 MHz, $CDCl_3$) δ 8.96 (s, 1H), 7.57 – 7.45 (m, 3H), 7.44 – 7.34 (m, 2H), 7.21 – 7.13 (m, 1H), 7.11 (d, *J* = 2.5 Hz, 1H), 6.78 (dd, *J* = 8.6, 2.5 Hz, 1H), 3.8 (s, 3H).

***N*-Phenyl-5-(trifluoromethyl)benzo[*d*]thiazol-2-amine (4d)**

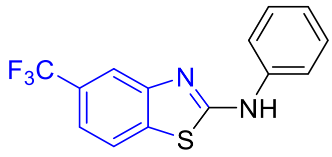

Following the general procedure, from 2-iodo-5-(trifluoromethyl)aniline (**2d**) and phenyl isothiocyanate (**3a**), compound **4d** was obtained in 63% yield (18.5 mg, 0.063 mmol) as a white solid after purification by flash column

chromatography (CyHx/EtOAc: 20/1 → 4/1). Spectroscopic data were consistent with the literature data for this compound.[18]

^{1}H NMR (300 MHz, $CDCl_3$) δ 7.86 (s, 1H), 7.76 – 7.67 (m, 2H), 7.57 – 7.49 (m, 2H), 7.46 – 7.36 (m, 3H), 7.24 – 7.13 (m, 1H).

Methyl 2-(phenylamino)benzo[*d*]thiazole-5-carboxylate (4e)

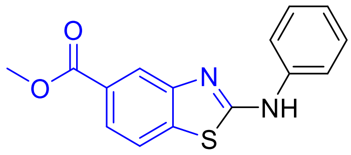

Following the general procedure, from methyl 3-amino-4-iodobenzoate (**2e**) and phenyl isothiocyanate (**3a**), compound **4e** was obtained in 70% yield (19.9 mg, 0.070 mmol) as a white solid after purification by flash column chromatography (CyHx/EtOAc: 20/1 → 4/1). Spectroscopic data were consistent with the literature data for this compound.[19]

^{1}H NMR (300 MHz, DMSO) δ 10.64 (s, 1H), 8.11 (s, 1H), 7.99 – 7.92 (m, 1H), 7.84 – 7.71 (m, 4H), 7.39 (t, J = 7.7 Hz, 2H), 7.12 – 7.02 (m, 1H), 3.89 (s, 3H).

5-Bromo-*N*-phenylbenzo[*d*]thiazol-2-amine (4f)

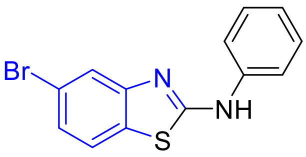

Following the general procedure, from methyl 5-bromo-2-iodoaniline (**2f**) and phenyl isothiocyanate (**3a**), compound **4f** was obtained in 53% yield (16.2 mg, 0.053 mmol) as a white solid after purification by flash column chromatography (CyHx/EtOAc: 20/1 → 4/1). Spectroscopic data were consistent with the literature data for this compound.[18]

^{1}H NMR (300 MHz, $CDCl_3$) δ 7.78 (d, J = 1.9 Hz, 1H), 7.57 – 7.45 (m, 3H), 7.44 – 7.37 (m, 2H), 7.29 (d, J = 1.9 Hz, 1H), 7.18 (t, J = 7.3 Hz, 1H).

5-Chloro-*N*-phenylbenzo[*d*]thiazol-2-amine (4g)

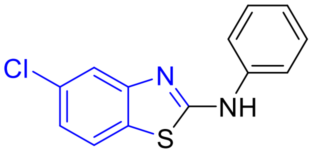

Following the general procedure, from methyl 5-chloro-2-iodoaniline (**2g**) and phenyl isothiocyanate (**3a**), compound **4g** was obtained in 54% yield (14.1 mg, 0.054 mmol) as a white solid after purification by flash column chromatography (CyHx/EtOAc: 20/1 → 9/1). Spectroscopic data were consistent with the literature data for this compound.[17]

^{1}H NMR (300 MHz, DMSO) δ 10.60 (s, 1H), 7.94 – 7.71 (m, 3H), 7.63 (d, J = 2.1 Hz, 1H), 7.37 (t, J = 7.9 Hz, 2H), 7.19 (dd, J = 8.4, 2.1 Hz, 1H), 7.05 (t, J = 7.4 Hz, 1H).

6-Methyl-*N*-phenylbenzo[*d*]thiazol-2-amine (4h)

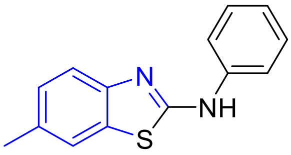

Following the general procedure, from methyl 2-iodo-4-methylaniline (**2h**) and phenyl isothiocyanate (**3a**), compound **4h** was obtained in 95% yield (22.8 mg, 0.095 mmol) as a white solid after purification by flash column chromatography (CyHx/EtOAc: 20/1 → 4/1). Spectroscopic data were consistent with the literature data for this compound.[16]

^{1}H NMR (300 MHz, $CDCl_3$) δ 7.56 – 7.46 (m, 3H), 7.45 – 7.35 (m, 3H), 7.18 – 7.10 (m, 2H), 2.42 (s, 3H).

6-Methoxy-*N*-phenylbenzo[*d*]thiazol-2-amine (4i)

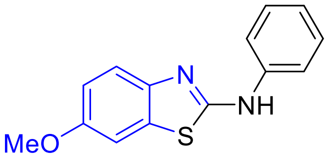

Following the general procedure, from methyl 2-iodo-4-methoxyaniline (**2i**) and phenyl isothiocyanate (**3a**), compound **4i** was obtained in 96% yield (24.6 mg, 0.096 mmol) as a purple solid after purification by flash column chromatography (CyHx/EtOAc: 20/1 → 9/1). Spectroscopic data were consistent with the literature data for this compound.[20]

^{1}H NMR (500 MHz, $CDCl_3$) δ 8.69 (s, 1H), 7.55 – 7.44 (m, 3H), 7.43 – 7.34 (m, 2H), 7.20 – 7.10 (m, 2H), 6.93 (dd, *J* = 8.8, 2.6 Hz, 1H), 3.83 (s, 3H).

6-fluoro-N-phenylbenzo[d]thiazol-2-amine (4j)

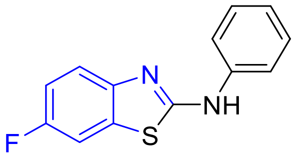

Following the general procedure, from 4-fluoro-2-iodoaniline (**2j**) and phenyl isothiocyanate (**3a**), compound **4j** was obtained in 89% yield (21.7 mg, 0.089 mmol) as a white solid after purification by flash column chromatography (CyHx/EtOAc: 3/1 → 1/3). Spectroscopic data were consistent with the literature data for this compound.[21]

^{1}H NMR (300 MHz, $CDCl_3$) δ 7.57 – 7.45 (m, 3H), 7.44 – 7.37 (m, 2H), 7.33 (dd, *J* = 8.1, 2.6 Hz, 1H), 7.21 – 7.13 (m, 1H), 7.06 (td, *J* = 9.0, 2.6 Hz, 1H).

6-Chloro-*N*-phenylbenzo[*d*]thiazol-2-amine (4k)

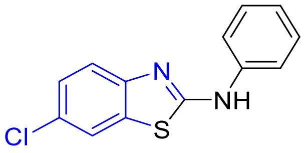

Following the general procedure, from 4-chloro-2-iodoaniline (**2k**) and phenyl isothiocyanate (**3a**), compound **4k** was obtained in 82% yield (21.4 mg, 0.082 mmol) as a white solid after purification by flash column chromatography (CyHx/EtOAc: 20/1 → 9/1). Spectroscopic data were consistent with the literature data for this compound.[16]

^{1}H NMR (300 MHz, DMSO) δ 10.54 (s, 1H), 7.93 (d, *J* = 2.2 Hz, 1H), 7.84 – 7.69 (m, 2H), 7.57 (d, *J* = 8.6 Hz, 1H), 7.42 – 7.31 (m, 3H), 7.15 – 6.98 (m, 1H).

_N_-Phenyl-6-(trifluoromethyl)benzo[_d_]thiazol-2-amine (4l)

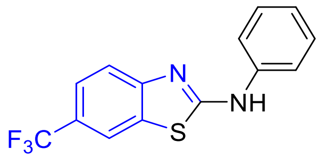

Following the general procedure, from 2-iodo-4-(trifluoromethyl)aniline (**2l**) and phenyl isothiocyanate (**3a**), compound **4l** was obtained in 56% yield (16.5 mg, 0.056 mmol) as a white solid after purification by flash column chromatography (CyHx/EtOAc: 20/1 → 4/1). Spectroscopic data were consistent with the literature data for this compound.[21]

^{1}H NMR (300 MHz, $CDCl_3$) δ 8.80 (s, 1H), 7.89 (dt, *J* = 1.6, 0.8 Hz, 1H), 7.66 – 7.56 (m, 2H), 7.55 – 7.40 (m, 4H), 7.24 – 7.20 (m, 1H).

4-Chloro-_N_-phenylbenzo[_d_]thiazol-2-amine (4m)

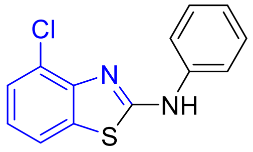

Following the general procedure, from 2-chloro-6-iodoaniline (**2m**) and phenyl isothiocyanate (**3a**), compound **4m** was obtained in 27% yield (7.0 mg, 0.027 mmol) as a white solid after purification by flash column chromatography (CyHx/EtOAc: 20/1 → 9/1). Spectroscopic data were consistent with the literature data for this compound.[22]

^{1}H NMR (300 MHz, DMSO) δ 10.70 (s, 1H), 7.89 – 7.73 (m, 4H), 7.47 – 7.32 (m, 4H), 7.14 (t, *J* = 7.9 Hz, 1H), 7.09 (t, *J* = 7.35 Hz 1H).

_N_-(4-Nitrophenyl)benzo[_d_]thiazol-2-amine (4n)

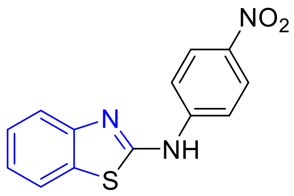

Following the general procedure, from 2-iodoaniline (**2a**) and 1-isothiocyanato-4-nitrobenzene (**3b**), compound **4n** was obtained in 96% yield (26.1 mg, 0.096 mmol) as a yellow solid after purification by flash column chromatography (CyHx/EtOAc: 9/1 → 3/1). Spectroscopic data were consistent with the literature data for this compound.[16]

^{1}H NMR (300 MHz, DMSO) δ 11.21 (s, 1H), 8.27 (d, *J* = 8.9 Hz, 2H), 8.01 (d, *J* = 8.9 Hz, 2H), 7.95 – 7.65 (m, 2H), 7.47 – 7.17 (m, 2H).

N-(3,5-Bis(trifluoromethyl)phenyl)benzo[d]thiazol-2-amine (4o)

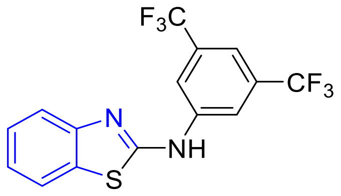

Following the general procedure, from 2-iodoaniline (**2a**) and 1-isothiocyanato-3,5-bis(trifluoromethyl)benzene (**3c**), compound **4o** was obtained in 88% yield (31.9 mg, 0.088 mmol) as a white-browned solid after purification by flash column chromatography (CyHx/EtOAc: 20/1). Spectroscopic data were consistent with the literature data for this compound.[18]

^{1}H NMR (300 MHz, $CDCl_3$) δ 8.75 (s, 1H), 8.08 (s, 2H), 7.74 – 7.66 (m, 2H), 7.59 (s, 1H), 7.41 (ddd, *J* = 8.0, 7.4, 1.3 Hz, 1H), 7.26 (td, *J* = 7.6, 1.1 Hz, 1H).

N-(4-Methoxyphenyl)benzo[d]thiazol-2-amine (4p)

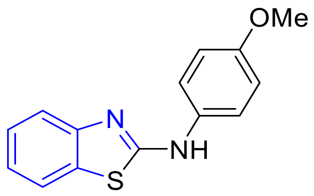

Following the general procedure, from 2-iodoaniline (**2a**) and 1-isothiocyanato-4-methoxybenzene (**3d**), compound **4p** was obtained in 90% yield (23.1 mg, 0,090 mmol) as a brown-oranged solid after purification by flash column chromatography (CyHx/EtOAc: 20/1 → 9/1). Spectroscopic data were consistent with the literature data for this compound.[16]

^{1}H NMR (300 MHz, $CDCl_3$) δ 9.06 (s, 1H), 7.59 – 7.54 (m, 1H), 7.49 – 7.38 (m, 3H), 7.32 – 7.28 (m, 1H), 7.10 (td, *J* = 7.5, 1.2 Hz, 1H), 7.01 – 6.93 (m, 2H) 3.86 (s, 3H).

6. NMR Spectra

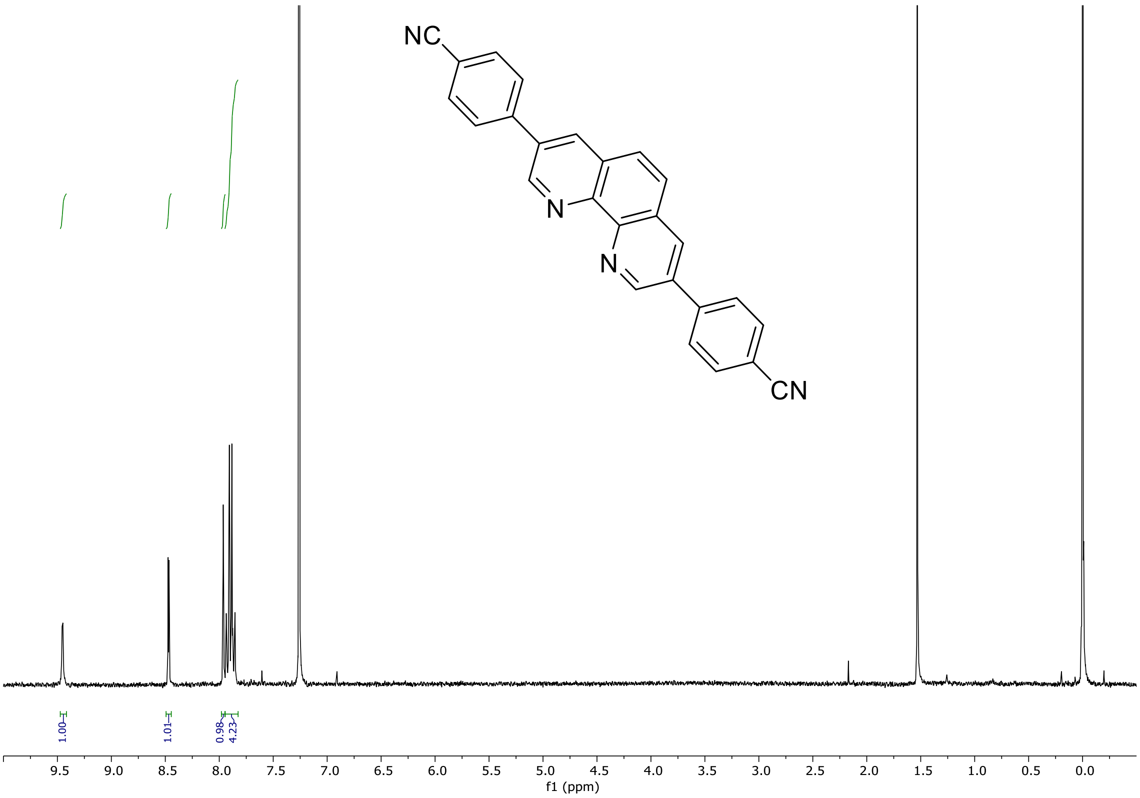

Figure S27. ^{1}H-NMR spectrum (300 MHz, 298 K, $CDCl_3$) of **1.**

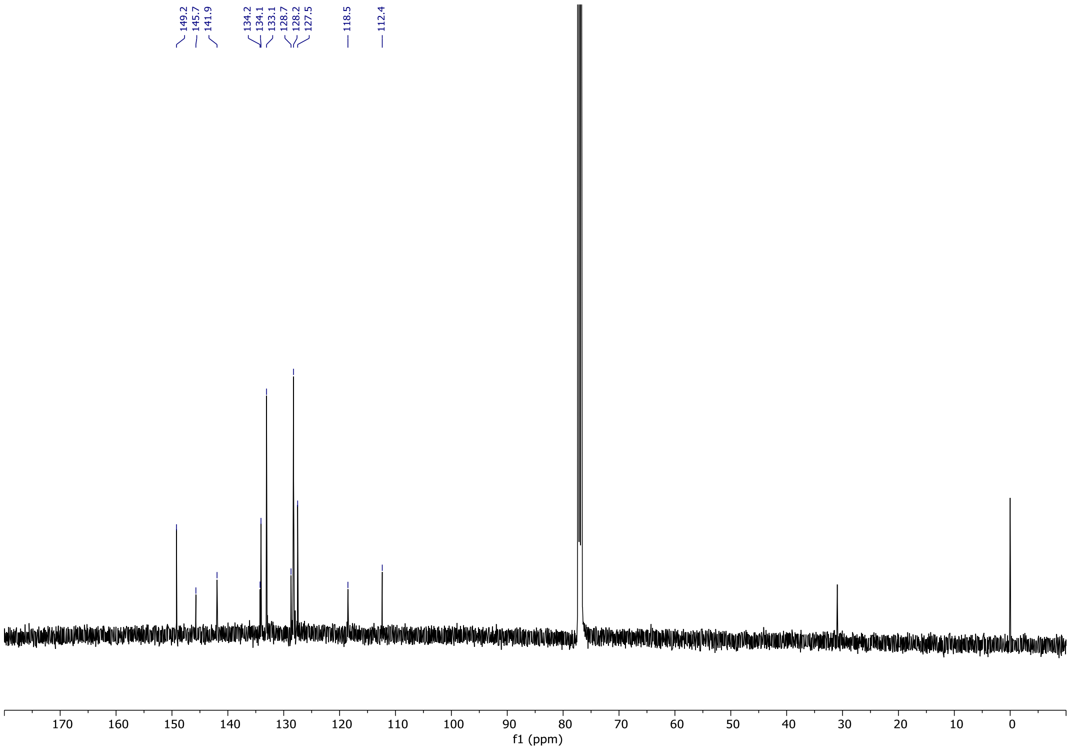

Figure S28. ^{13}C-NMR spectrum (126 MHz, 298 K, $CDCl_3$) of **1.**

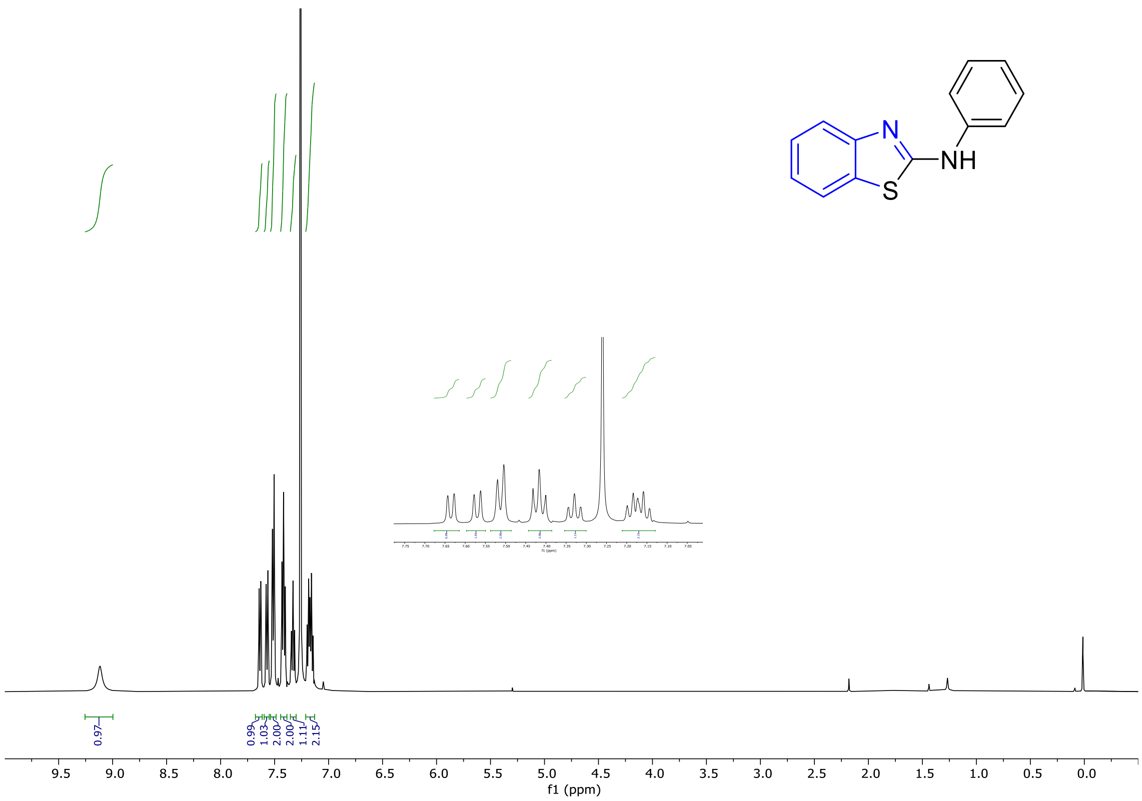

Figure S29. ^{1}H NMR spectrum (300 MHz, 298 K, $CDCl_3$) of **4a**.

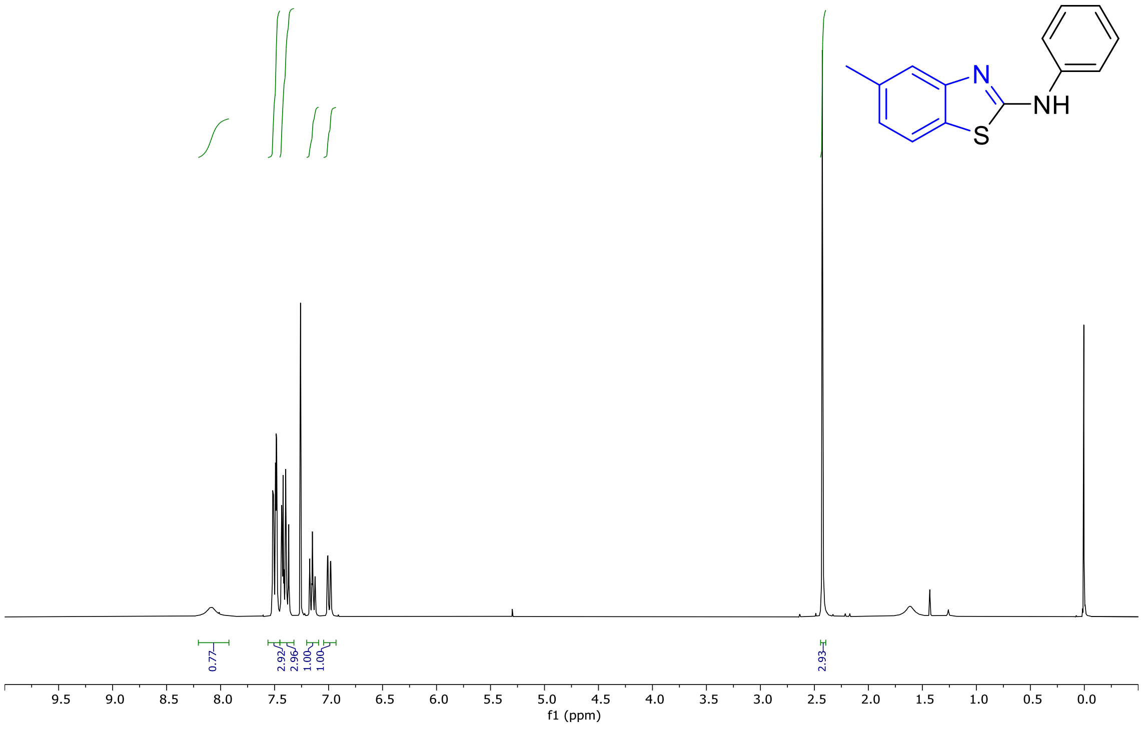

Figure S30. ^{1}H NMR spectrum (300 MHz, 298 K, $CDCl_3$) of **4b**.

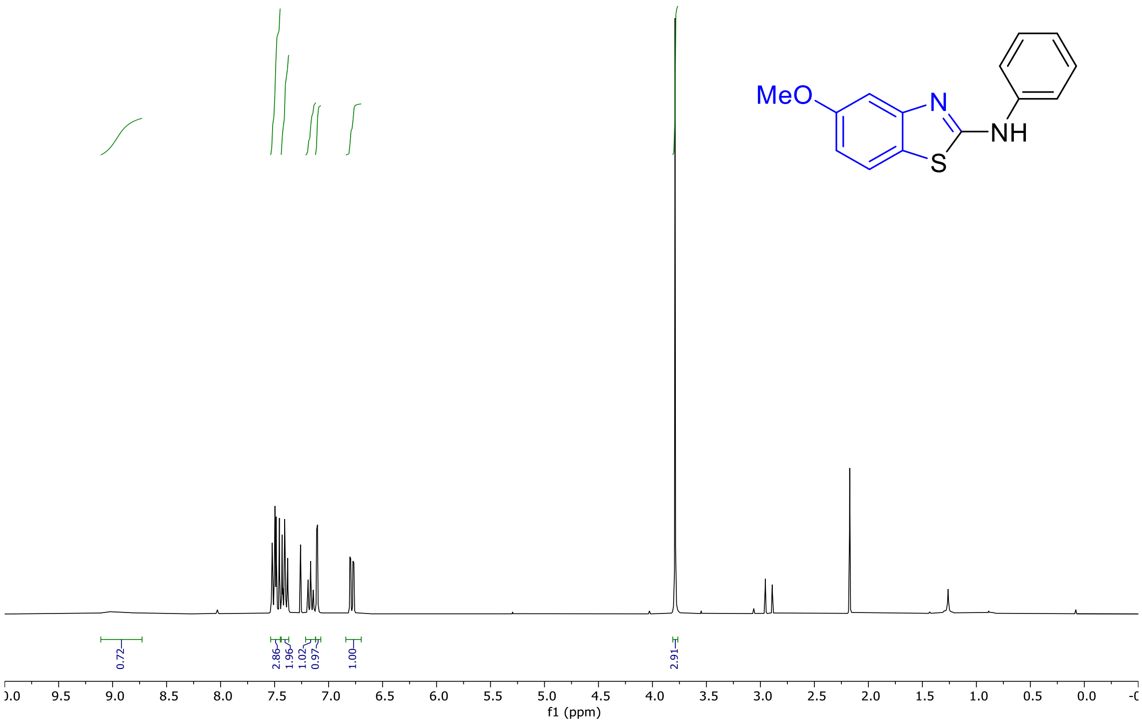

Figure S31. ^{1}H NMR spectrum (300 MHz, 298 K, $CDCl_3$) of **4c**.

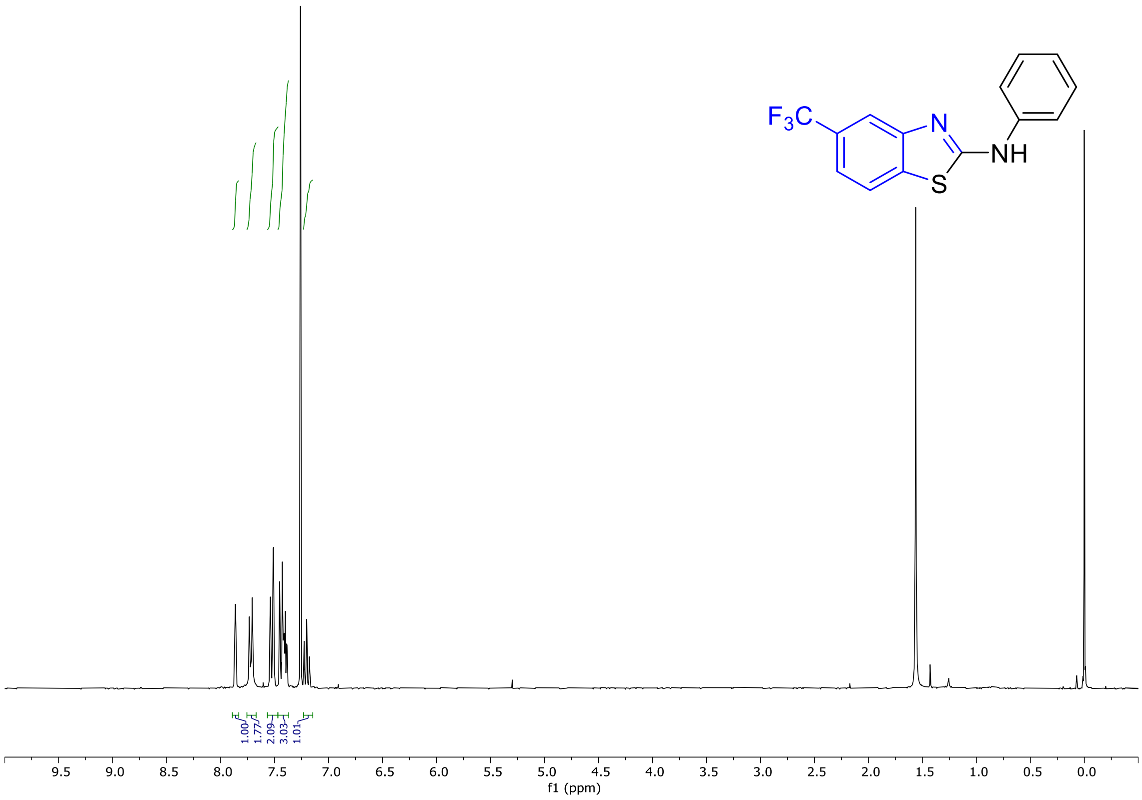

Figure S32. ^{1}H NMR spectrum (300 MHz, 298 K, $CDCl_3$) of **4d**.

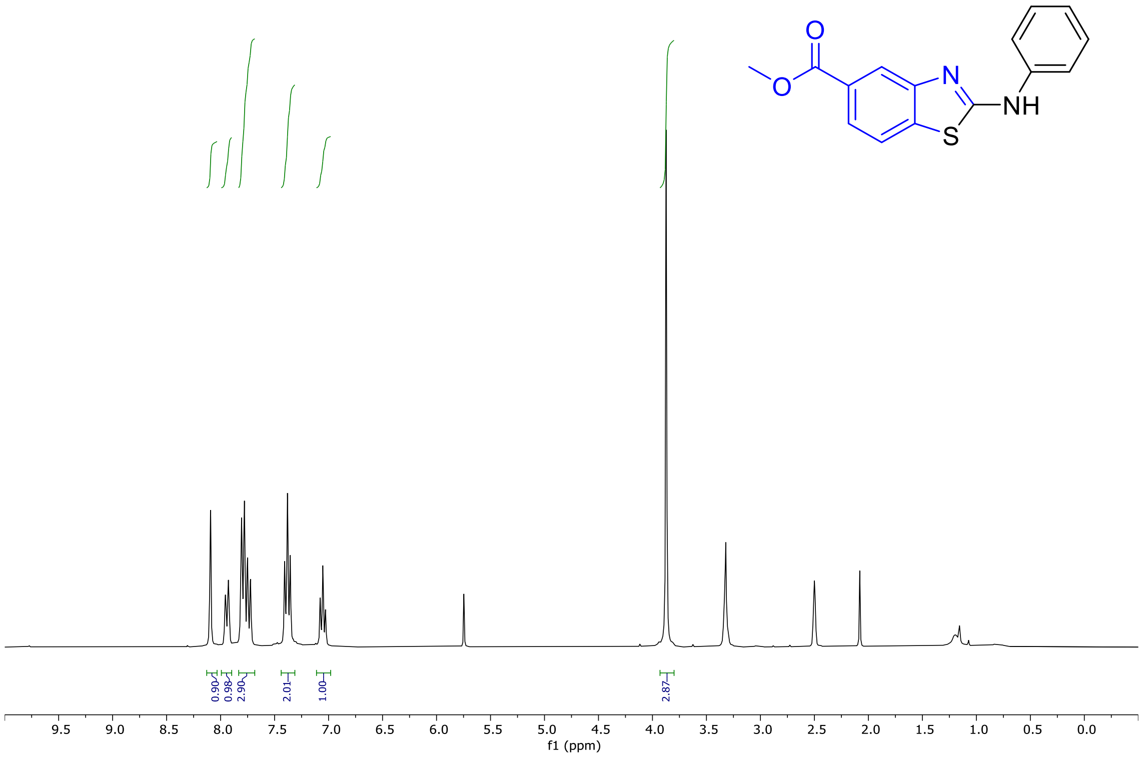

Figure S33. ^{1}H NMR spectrum (300 MHz, 298 K, DMSO-d6) of **4e**.

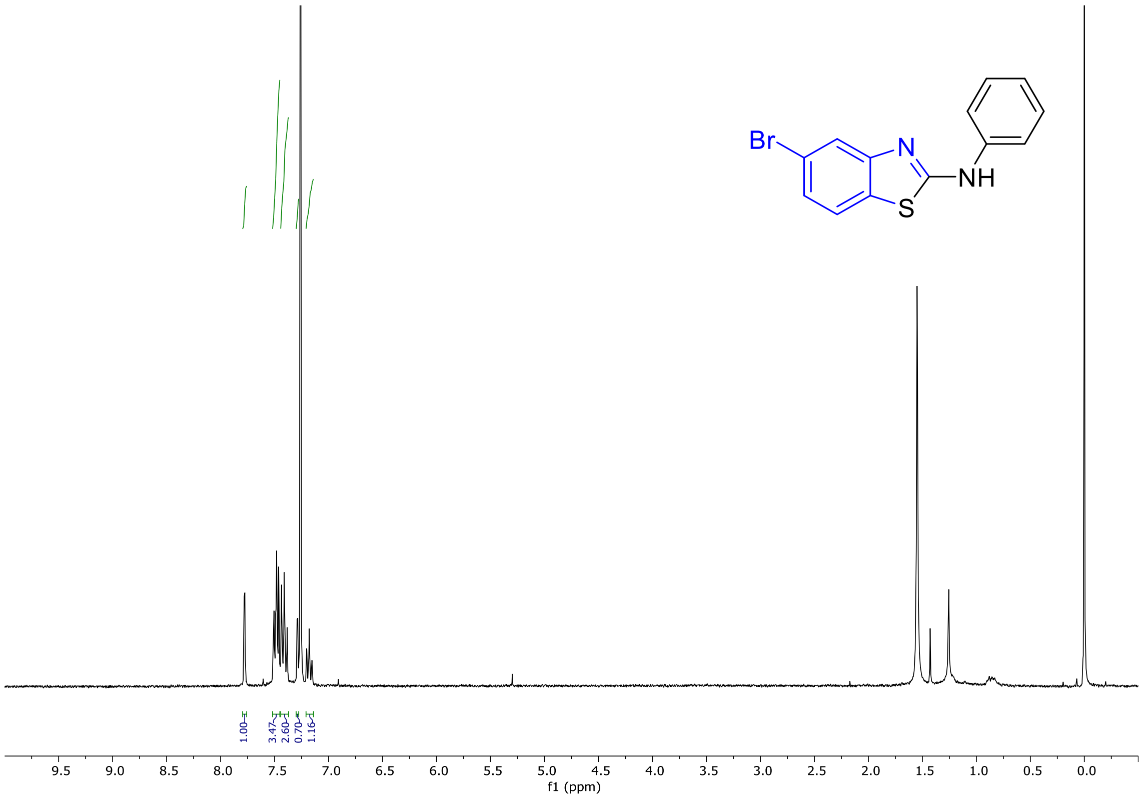

Figure S34. ^{1}H NMR spectrum (300 MHz, 298 K, $CDCl_3$) of **4f**.

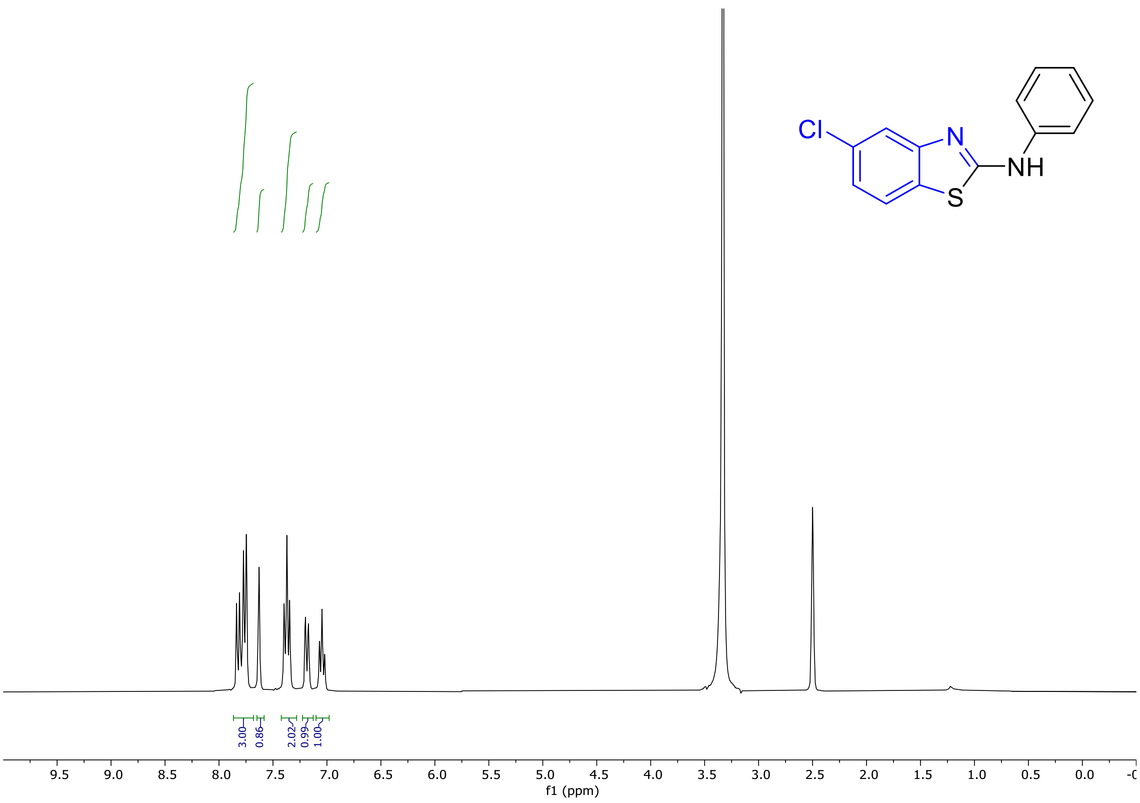

Figure S35. ^{1}H NMR spectrum (300 MHz, 298 K, DMSO-d6) of **4g**.

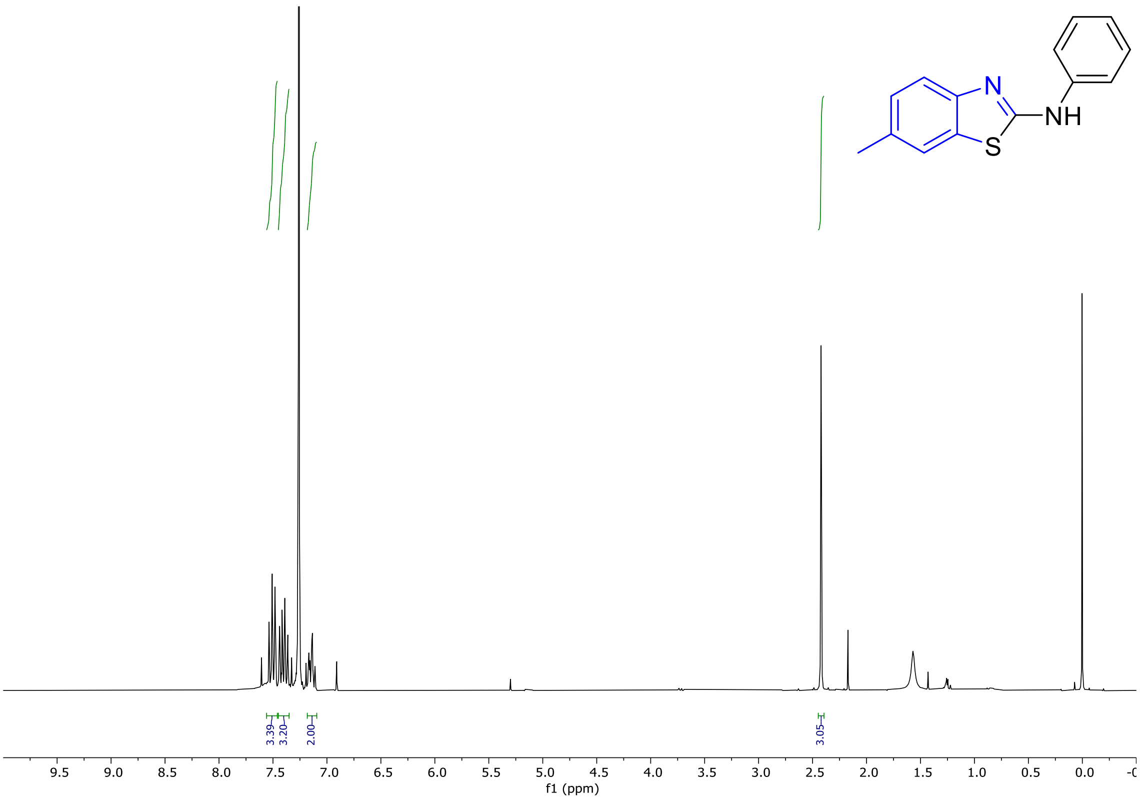

Figure S36. ^{1}H NMR spectrum (300 MHz, 298 K, $CDCl_3$) of **4h**.

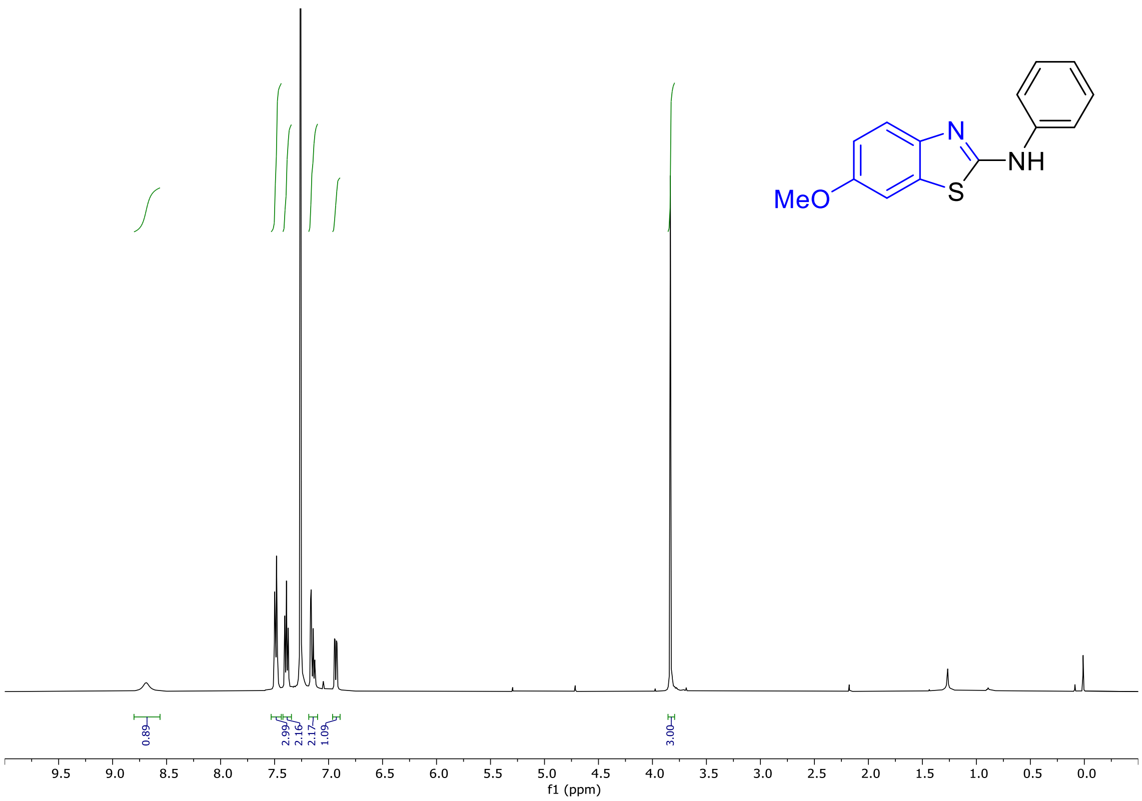

Figure S37. ^{1}H NMR spectrum (300 MHz, 298 K, $CDCl_3$) of **4i**.

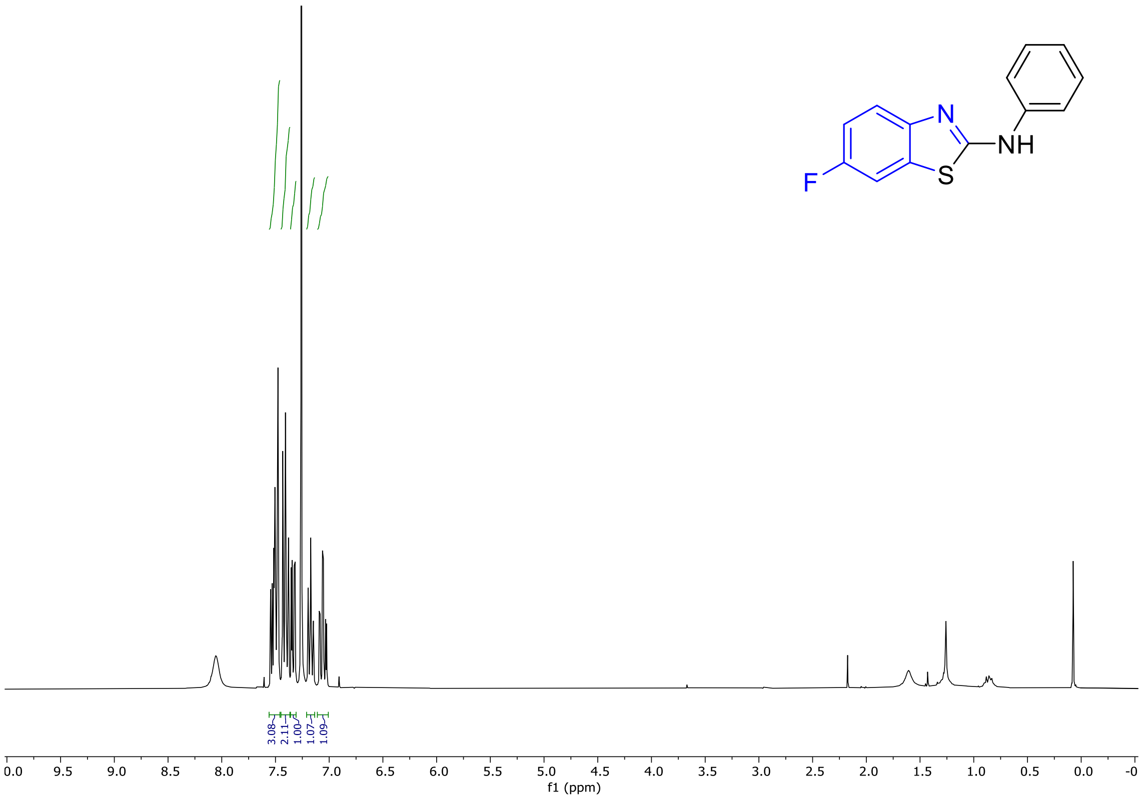

Figure S38. ^{1}H NMR spectrum (300 MHz, 298 K, $CDCl_3$) of **4j**.

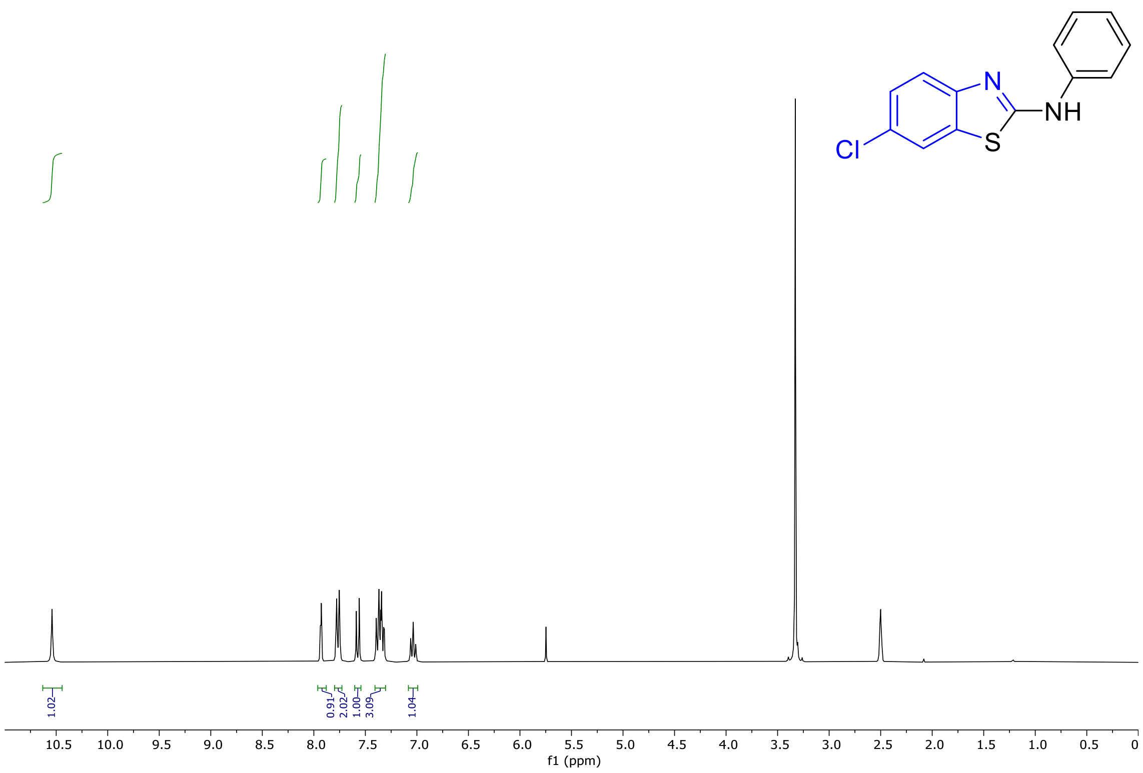

Figure S39. ^{1}H NMR spectrum (300 MHz, 298 K, DMSO-d6) of **4k**.

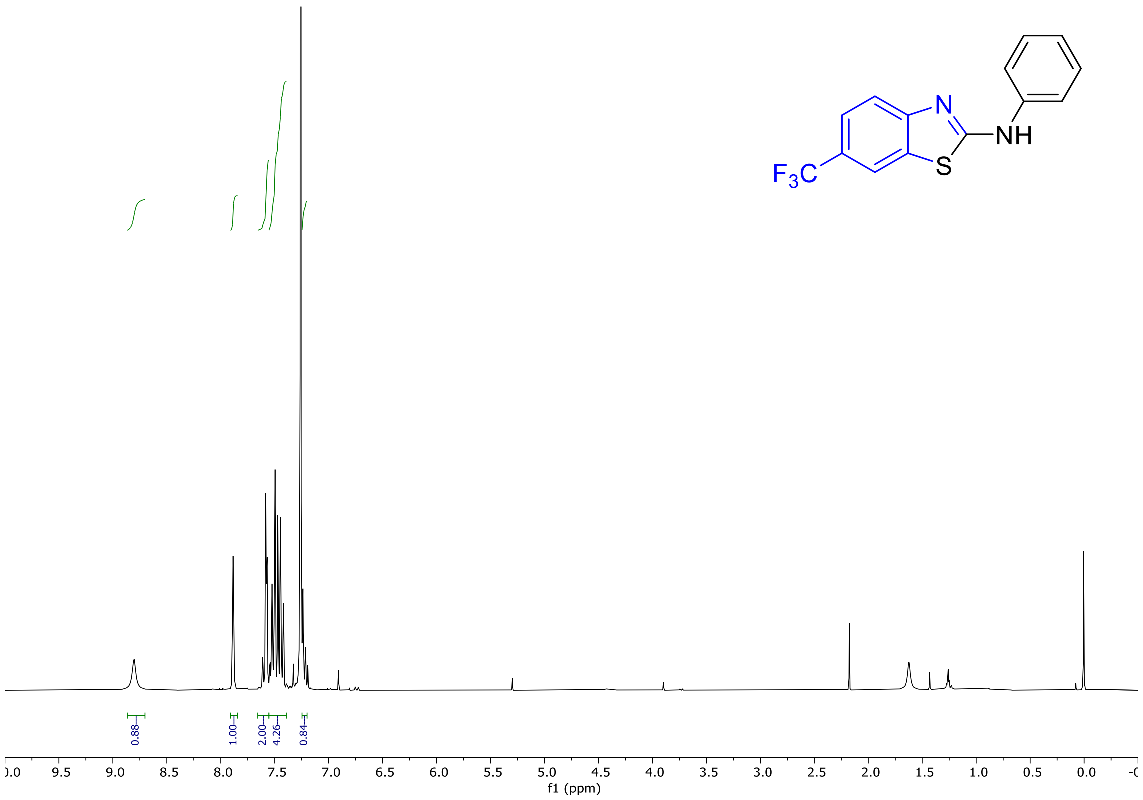

Figure S40. ^{1}H NMR spectrum (300 MHz, 298 K, $CDCl_3$) of **4l**.

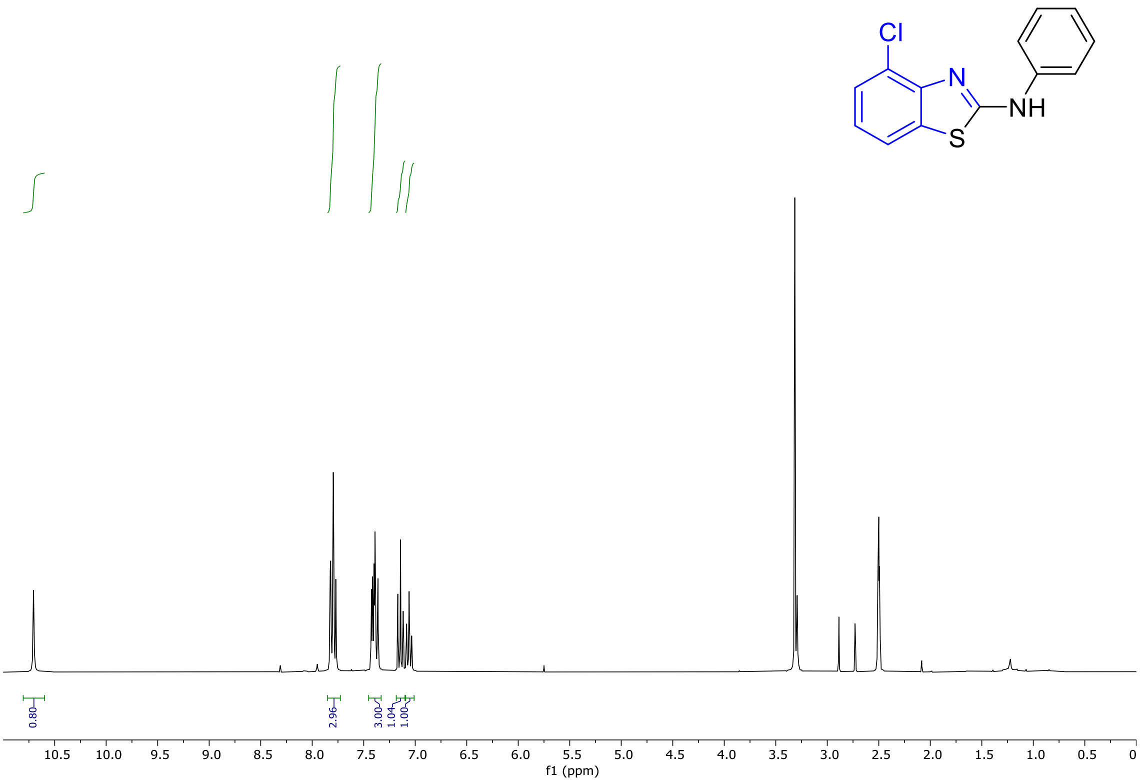

Figure S41. ^{1}H NMR spectrum (300 MHz, 298 K, DMSO-d6) of **4m**.

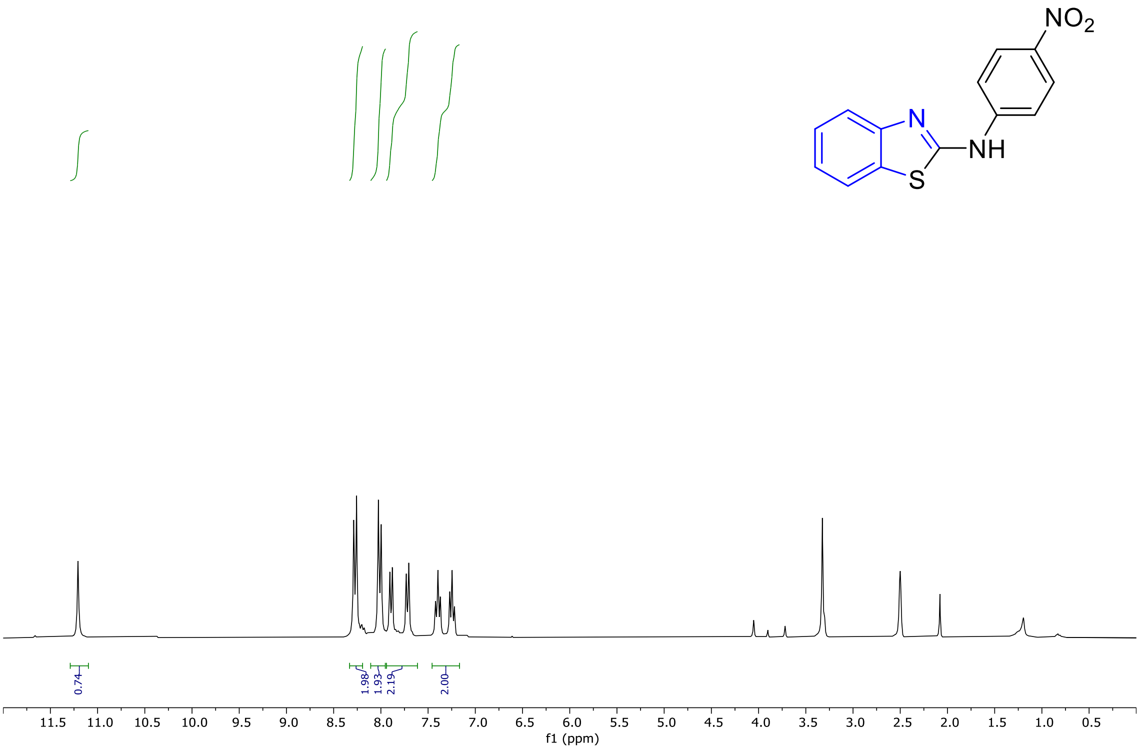

Figure S42. ^{1}H NMR spectrum (300 MHz, 298 K, DMSO-d6) of **4n**.

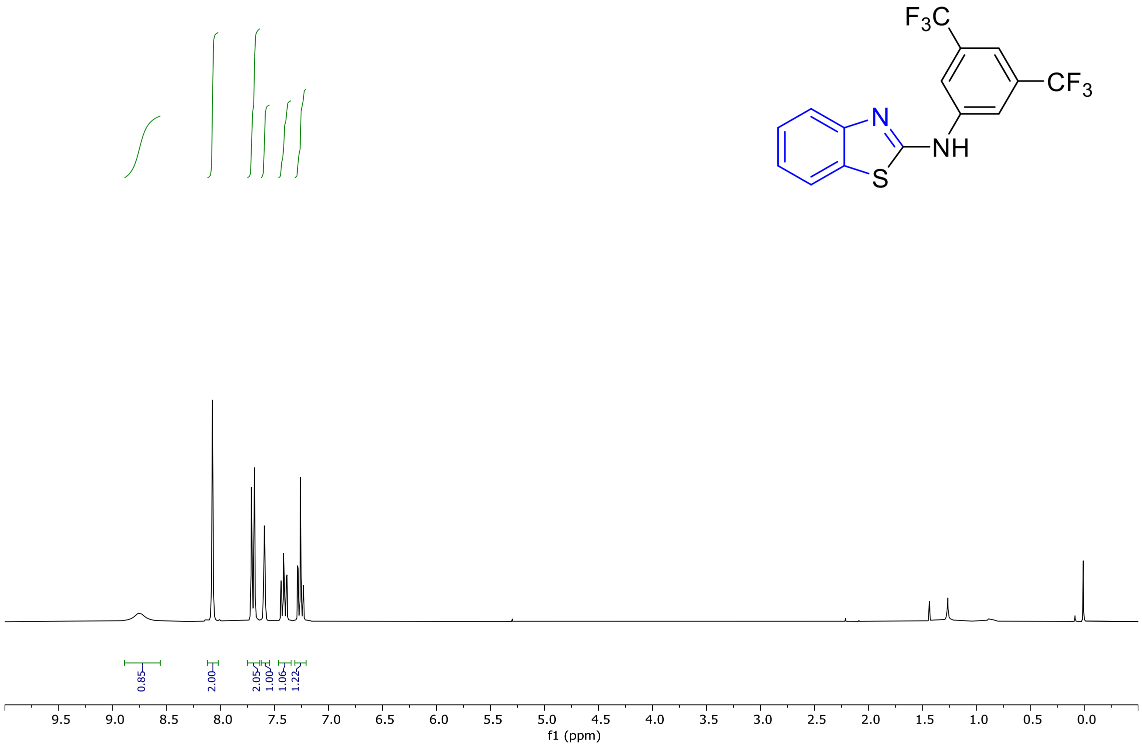

Figure S43. ^{1}H NMR spectrum (300 MHz, 298K, $CDCl_3$) of **4o**.

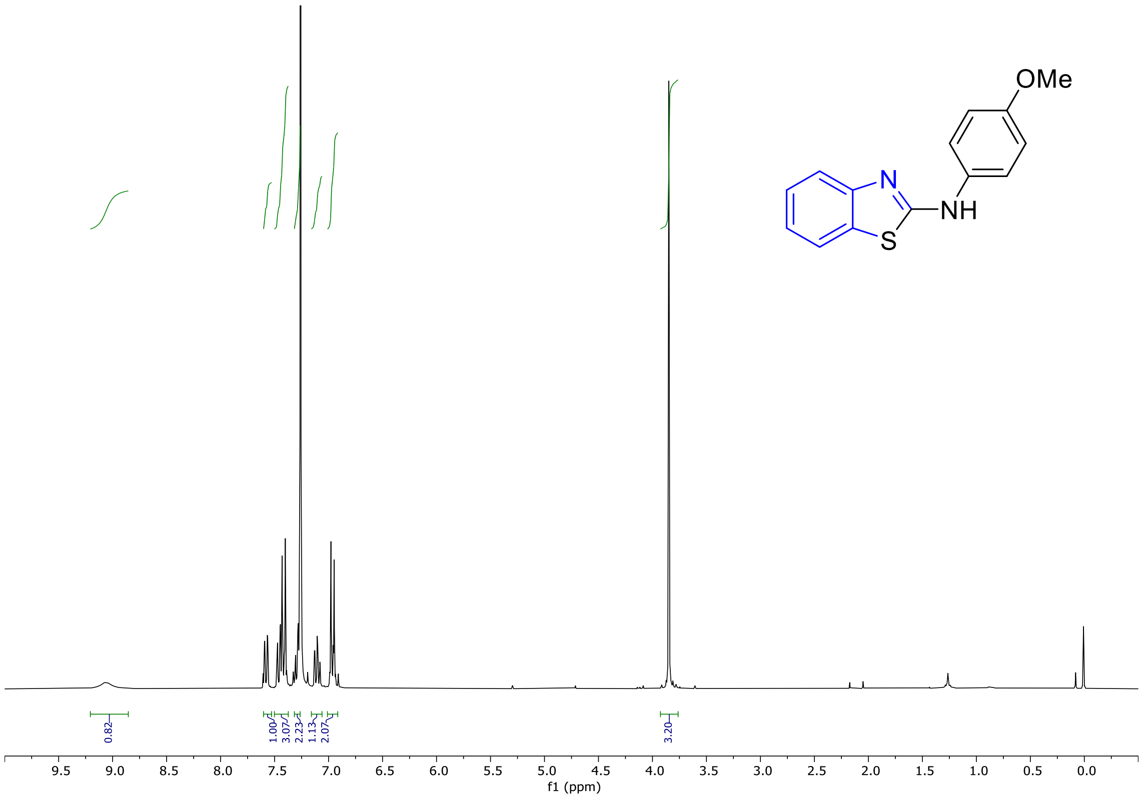

Figure S44. ^{1}H NMR spectrum (300 MHz, 298K, $CDCl_3$) of **4p**.

7. References

1. J. Verbeeck, G. Bertoni, Deconvolution of core electron energy loss spectra. *Ultramicroscopy* **2009**, *109*, 1343–1352.
2. J. J. de Rooi, N. M. van der Pers, R. W. A. Hendrikx, R. Delhez, A. J. Böttger, P. H. C. Eilers, Smoothing of X-ray diffraction data and $K\alpha_2$ elimination using penalized likelihood and the composite link model. *J. Appl. Cryst.* **2014**, *47*, 852–860.
3. S. Tougaard, Practical algorithm for background subtraction. *Surf. Sci*. **1989**, *216*, 343–360.
4. Doniach, S.; Sunjic, M. *J. Phys. C: Solid State Phys.* **1970**, *3*, 285.
5. M. P. Seah, W. A. Dench, Quantitative electron spectroscopy of surfaces: A standard data base for electron inelastic mean free paths in solids. *Surf. Interf. Anal.* **1979,** *1*, 2–11.
6. C. D. Wagner, L. E. Davis, M. V. Zeller, J. A. Taylor, R. H. Raymond. L. H. Gale, Empirical atomic sensitivity factors for quantitative analysis by electron spectroscopy for chemical analysis. *Surf. Interface Anal.*, **1981**, *3*, 211–225.
7. D. Briggs, M. P. Seah, Practical surface analysis, 2 nd ed., vol I, auger and X-ray photoelectron spectroscopy. John Wiley & Sons, New York, **1990**.
8. Yan, J.; Li, P.; Wang, L. *Tetrahedron*, **2011**, *67*, 5543–5549.
9. Xiao, R.; Hao, W.; Ai, J.; Cai, M.-Z. *J. Org. Chem.* **2012**, *705*, 4178–4184.
10. Liu, J.; Zhang, X.; Yang, J.; Wang, L. *Appl. Organometal. Chem.* **2014**, *28*, 198–203.
11. Gaddam, S.; Kasireddy, H.-R.; Konkala, K.; Katla, R.; Durga, N-Y-V. *Chin. Chem. Lett.* **2014**, *25*, 732–736.
12. Azizi, K.; Karimi, M.; Heydari, A. *Tetrahedron Lett.* **2015**, *56*, 812–816.
13. Moghadam, F.-K.; Jarrah, N.; Mashayekh-Salehi, A.; Ghanbaripour, R. *Synlett* **2016**, *27*, 1665–1668.
14. Yue, J.-Y.; Wang, L.; Zhang, L.; Ma, Y.; Yang, P. Tang, B. *Micropor. Mesopor. Mat*. **2020**, *305*, 110313.
15. Wang, J.-C.; Yu, Z.-G.; Yang, W.-T.; Du, J.-Q, Chen, Z.; Kan, J.-L.; Dong, Y.; Dong, Y.-B. *Chem. Plus. Chem.* **2024**, *89*, e202300494.
16. Guo, Y.-J.; Tang, R.-Y.; Zhong, P.; Li, J.-H. *Tetrahedron Lett.* **2010**, *51*, 649–652.

17. Boddapati, S. N. M.; Kurmarayuni, C. M.; Mutchu, B. R.; Tamminana, R.; Bollikolla, H. B. *Org. Biomol. Chem.* **2018**, *16*, 8267-8272.

18. Nguyen, D. K.; Pham, T. D. B.; Tran, G. T. H.; Nguyen, P. D.; Chau, T. K.; Ong, K. T. N.; Nguyen, A. T.; Nguyen, T. T. *Eur. J. Org. Chem.* **2025**, *28*, e202401080.

19. Zhang, W.; Yue, Y.; Yu, D.; Song, L.; Xu, Y.-Y.; Tian, Y.-J.; Guo, Y.-J. *Adv. Synth. Catal.* **2012**, *354*, 2283.

20. Cheng, H.; Zhu, Y.-Q.; Liu, P.-F.; Yang, K.-Q.; Yan, J.; Sang, W.; Tang, X.-S.; Zhang, R.; Chen, C. *J. Org. Chem.* **2021**, *86*, 10288–10302.

21. Y.L. Sun, Y. Zhang, X.H. Cui, W. Wang, *Adv. Synth. Catal.* **2011**, *353*, 1174–1178.

22. Zhang, X.; Jia, X.; Wang, J.; Fan, X. *Green Chem.* **2011**, *13*, 413–418.
